# New Hybrids of 4-Amino-2,3-polymethylene-quinoline and *p*-Tolylsulfonamide as Dual Inhibitors of Acetyl- and Butyrylcholinesterase and Potential Multifunctional Agents for Alzheimer’s Disease Treatment

**DOI:** 10.3390/molecules25173915

**Published:** 2020-08-27

**Authors:** Galina F. Makhaeva, Nadezhda V. Kovaleva, Natalia P. Boltneva, Sofya V. Lushchekina, Tatiana Yu. Astakhova, Elena V. Rudakova, Alexey N. Proshin, Igor V. Serkov, Eugene V. Radchenko, Vladimir A. Palyulin, Sergey O. Bachurin, Rudy J. Richardson

**Affiliations:** 1 Institute of Physiologically Active Compounds, Russian Academy of Sciences, 142432 Chernogolovka, Russia; gmakh@ipac.ac.ru (G.F.M.); kovalevanv@ipac.ac.ru (N.V.K.); boltneva@ipac.ac.ru (N.P.B.); sofya.lushchekina@gmail.com (S.V.L.); rudakova@ipac.ac.ru (E.V.R.); proshin@ipac.ac.ru (A.N.P.); serkoviv@mail.ru (I.V.S.); genie@qsar.chem.msu.ru (E.V.R.); vap@qsar.chem.msu.ru (V.A.P.); bachurin@ipac.ac.ru (S.O.B.); 2 Emanuel Institute of Biochemical Physics, Russian Academy of Sciences, 119334 Moscow, Russia; astakhova1967.t@yandex.ru; 3 Department of Chemistry, Lomonosov Moscow State University, 119991 Moscow, Russia; 4 Department of Environmental Health Sciences, University of Michigan, Ann Arbor, MI 48109, USA; 5 Department of Neurology, University of Michigan, Ann Arbor, MI 48109, USA; 6 Center of Computational Medicine and Bioinformatics, University of Michigan, Ann Arbor, MI 48109, USA

**Keywords:** 4-amino-2,3-polymethylene-quinoline, acetylcholinesterase (AChE), ADMET, Alzheimer’s disease (AD), butyrylcholinesterase (BChE), molecular docking, *p*-tolylsulfonamide

## Abstract

New hybrid compounds of 4-amino-2,3-polymethylene-quinoline containing different sizes of the aliphatic ring and linked to *p*-tolylsulfonamide with alkylene spacers of increasing length were synthesized as potential drugs for treatment of Alzheimer’s disease (AD). All compounds were potent inhibitors of acetylcholinesterase (AChE) and butyrylcholinesterase (BChE) with selectivity toward BChE. The lead compound 4-methyl-N-(5-(1,2,3,4-tetrahydro-acridin-9-ylamino)-pentyl)-benzenesulfonamide (**7h**) exhibited an IC_50_ (AChE) = 0.131 ± 0.01 µM (five times more potent than tacrine), IC_50_(BChE) = 0.0680 ± 0.0014 µM, and 17.5 ± 1.5% propidium displacement at 20 µM. The compounds possessed low activity against carboxylesterase, indicating a likely absence of unwanted drug-drug interactions in clinical use. Kinetics studies were consistent with mixed-type reversible inhibition of both cholinesterases. Molecular docking demonstrated dual binding sites of the conjugates in AChE and clarified the differences in the structure-activity relationships for AChE and BChE inhibition. The conjugates could bind to the AChE peripheral anionic site and displace propidium, indicating their potential to block AChE-induced β-amyloid aggregation, thereby exerting a disease-modifying effect. All compounds demonstrated low antioxidant activity. Computational ADMET profiles predicted that all compounds would have good intestinal absorption, medium blood-brain barrier permeability, and medium cardiac toxicity risk. Overall, the results indicate that the novel conjugates show promise for further development and optimization as multitarget anti-AD agents.

## 1. Introduction

Alzheimer’s disease (AD) is a common neurodegenerative disorder in the elderly that is manifested by progressive loss of memory and cognitive functions, which inevitably leads to disability and death [1]. The exact molecular mechanisms involved in AD initiation and progression are still unclear. However, it is obvious now that AD is a complex, multifactorial disorder and that advanced age is the main risk factor [2,3]. Several conditions are known to participate in the associated neurodegeneration. Accumulation of amyloid β peptide (Aβ) deposits, abnormal modification and accumulation of the tau protein accompanied by oxidative stress in the brain lead to synaptic dysfunction and neurodegeneration [4,5]. These processes most severely affect the cholinergic system and result in a progressive decrease in levels of the neurotransmitter acetylcholine (ACh) [6], which in turn produces the memory and cognitive deficits characteristic for patients with AD [7].

The anticholinesterase drugs donepezil (Aricept), galantamine (Reminyl), and rivastigmine (Exelon) are aimed at compensating the deficiency of ACh and are currently the main pharmacotherapeutic group used for the symptomatic treatment of AD [8,9,10,11]. These agents help to restore brain ACh levels by inhibiting cholinesterases, thereby increasing the duration of ACh action on postsynaptic receptors and enhancing cholinergic transmission. The brain contains two major forms of cholinesterases: acetylcholinesterase (AChE, EC 3.1.1.7) and butyrylcholinesterase (BChE, EC 3.1.1.8), which terminate cholinergic neurotransmission by the hydrolysis of ACh [12]. In a healthy brain, acetylcholine is predominantly (80%) hydrolyzed by AChE, whereas BChE plays a supplementary role serving as a co-regulator of AChE activity [13]. The cholinergic therapy of AD was originally directed primarily toward the inhibition of AChE [11,14]. However, with progression of AD, the activity of AChE decreases while the activity of BChE gradually increases [13,15,16]. This enlarges the significance of BChE as an additional therapeutic target for reducing the cholinergic deficiency inherent in AD [17,18,19,20]. Selective inhibitors of BChE can increase the brain level of acetylcholine and enhance cognitive functions in rodents without the usual side effects typical of AChE inhibitors [17,18,21]. It is believed that compounds inhibiting both cholinesterases increase the efficiency of treatment [19,20,22]. However, although these medications can temporarily reduce the development of AD symptoms, they cannot stop the progression of neurodegeneration [8,14].

The formation of β-amyloid (Aβ) deposits is typical of the early pathogenesis in AD. Many lines of evidence suggest that both AChE and BChE, which are localized within Aβ plaques in the AD brain, are related to amyloid plaque formation and thus contribute to the neuropathology of the disease [23,24,25]. AChE has proaggregant properties toward β-amyloid via involvement of the AChE peripheral anionic site (PAS) located at the rim of the gorge, which interacts with soluble β-amyloid peptides, thus promoting their aggregation [24,26,27,28,29]. It has been found that PAS ligands, such as propidium, block AChE-induced Aβ aggregation by physically hampering Aβ binding to the enzyme surface [26,27,30]. Moreover, dual-binding molecules, which interact with both the catalytic active site (CAS) and the PAS of AChE, can inhibit AChE activity and block its amyloidogenic properties. Such compounds not only can cause alleviation of the cognitive impairment of AD individuals via increasing the levels of acetylcholine but also can play a role as disease-modifying agents that can delay the formation of Aβ plaques [28,31,32,33,34]. BChE is also involved in the formation and/or maturation of Aβ plaques and thereby contributes to the pathogenesis of AD [15,35]. Therefore, inhibitors of both cholinesterases are of interest from the point of view of anti-amyloid strategy.

Currently, the multifactorial nature of AD has changed the approach to searching for effective drugs from the concept of single-target molecules to the design of multi-target agents that can simultaneously modulate multiple biological targets involved in disease pathogenesis [3,36,37,38,39]. Cholinesterases are still the most promising targets for ameliorating cognitive dysfunctions in AD. Accordingly, in the design of multi-target drugs, a cholinesterase inhibitor is selected as one of the pharmacophores. A second pharmacophore is then attached via a spacer to the cholinesterase inhibitor [40,41,42,43,44]. Such hybrid molecules expand the range of pharmacological activities of a parent anticholinesterase compound by adding antiaggregant, antioxidant, and other desirable properties [45,46,47,48,49,50].

Tacrine is the first drug approved for AD treatment. It has a high affinity for the AChE CAS and is widely employed as a pharmacophore to create multifunctional cholinesterase inhibitors possessing additional neuroprotective and disease-modifying properties [46,51,52,53,54,55,56,57].

**Figure 1:**
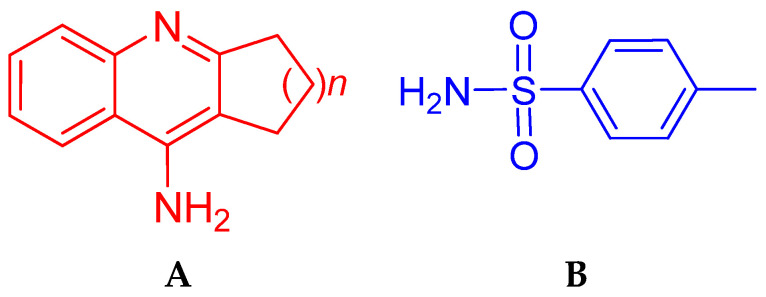
Structures of pharmacophores: (**A**) 4-amino-2,3-polymethylene-quinolines; (**B**) *p*-tolylsulfonamide.

In the present work, we used 4-amino-2,3-polymethylene-quinoline with different sizes of the aliphatic ring as the main anticholinesterase pharmacophore (Figure 1A); the homologue with *n* = 2 is tacrine.

The aromatic *p*-tolylsulfonamide moiety (Figure 1B) was used as our second pharmacophore. Sulfonamides are widely utilized in various pharmaceutical applications due to their therapeutic versatility that encompasses antibacterial, antiviral, antimalarial, antifungal, anticancer, antidepressant, and other properties [58,59]. They are also among the most effective and highly investigated derivatives in the field of carbonic anhydrases inhibition and related clinical applications [60,61,62]. Recently, publications have appeared on the presence of inhibitory activity against AChE and BChE in various sulfonamide derivatives [63,64,65,66,67]. Sulfonamide-containing selective and reversible human BChE inhibitors with nanomolar potency were developed that improved learning and memory functions in mouse models [21]. Moreover, antioxidant activity has been shown for certain sulfonamide derivatives [65,66,68,69]. Several sulfonamide-containing compounds exhibited promising Aβ self-assembly and cholinesterase inhibition, and in parallel showed good free radical scavenging properties [63].

The incorporation of a sulfonamide fragment into hybrid structures for anti-AD drugs has likewise been demonstrated. For example, a series of tacrine derivatives containing a sulfonamide group was synthesized; the compounds exhibited potent inhibitory activity on both cholinesterases and were shown to have antioxidant properties [69]. In addition, quinoline-sulfonamide hybrids have been developed as potential multifunctional therapeutic agents for AD with high anti-AChE and anti-BChE activity; the lead compound in this series was shown to improve hippocampal-dependent working memory in a rat model [70].

Herein, we have synthesized new hybrid compounds of 4-amino-2,3-polymethylene-quinoline containing different sizes of the aliphatic ring and linked to *p*-tolylsulfonamide using alkylene spacers of increasing length. We investigated the action of the synthesized compounds on enzyme targets of the cholinergic nervous system using as surrogates human erythrocyte acetylcholinesterase (EC 3.1.1.7, AChE) and equine serum butyrylcholinesterase (EC 3.1.1.8, BChE), along with a structurally related enzyme, porcine liver carboxylesterase (EC 3.1.1.1, CES). We also studied the ability of our novel conjugates to bind to the PAS of AChE from *Electrophorus electricus* (*Ee*AChE) and competitively displace propidium iodide from this site as a measure of their potential ability to block AChE-induced aggregation of β-amyloid. Enzyme kinetics studies were used to ascertain the mechanism of inhibition, and molecular docking was employed to provide insights into the binding modes of conjugates in the AChE and BChE active sites. We also measured the radical-scavenging activity of the conjugates in the ABTS assay. Finally, to assess the potential pharmacokinetic properties of the new compounds, we computationally estimated their profiles for absorption, distribution, metabolism, excretion, and toxicity (ADMET). Some preliminary results have been published earlier [71].

## 2. Results and Discussion

### 2.1. Chemistry

**Figure 2:**
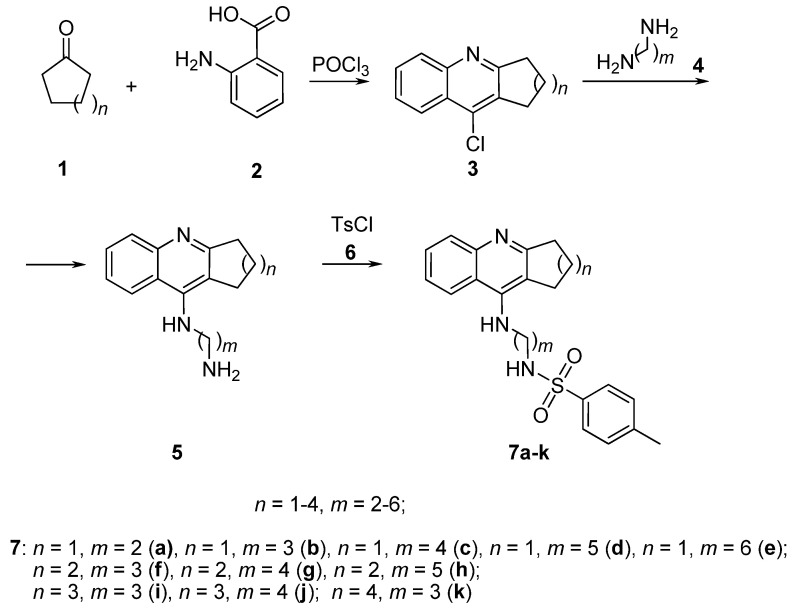
Synthesis of conjugates of 4-amino-2,3-polymethylene-quinolines and *p*-tolylsulfonamide **7**.

We have developed a method for the synthesis of hybrid multi-target compounds **7a**–**k** based on 4-amino-2,3-polymethylene-quinolines as the first pharmacophore and *p*-tolylsulfonamide as the second pharmacophore. Previously, we synthesized such compounds **7b**,**f**,**i**,**k** in which the quinoline and sulfonamide pharmacophores were combined into one molecule with a diaminopropane linker, in the form of hydrochlorides [71]. Here, we devised a procedure for the synthesis of target conjugates in the form of free bases. As shown in Figure 2, these compounds were obtained by the reaction of aminoquinolines **5** with tosyl chloride **6** in the presence of triethylamine. The aminoquinolines **5** needed for this were synthesized by the following method. First, by boiling anthranilic acid (**2**) with ketones **1** (cyclopentanone, cyclohexanone, cycloheptanone or cyclooctanone) in phosphorus oxychloride, chlorine derivatives **3** were obtained. Then, derivatives **3** were condensed with diamines **4** according to a published procedure [72] to afford the necessary aminoquinolines **5**.

Thus, conjugates **7** with different sizes of the aliphatic ring of 4-amino-2,3-polymethylene-quinolines and various lengths of the alkyl chain linking the two pharmacophores have been synthesized. The size of the aliphatic ring and the length of the alkyl spacer for each compound **7** are shown in Table 1.

### 2.2. Inhibition Studies of AChE, BChE and CES. Structure-Activity Relationships

For all conjugates **7**, we determined their esterase profile, i.e., the relative ability to inhibit several related serine esterases – AChE, BChE, and CES. This approach makes it possible to estimate both the primary pharmacological effects of the tested compounds (the inhibition of AChE and BChE) and their possible unwanted side effects (the inhibition of CES that hydrolyzes numerous ester-containing drugs) [73,74,75,76,77]. AChE from human erythrocytes was used along with two enzymes of non-human origin: equine serum BChE and porcine liver CES. These sources of BChE and CES were used because of their relatively low cost, high sequence identity to human enzymes [73,74,76], and the exploratory character of this work.

The inhibitory activities of our conjugates against the esterases were characterized as IC_50_ values, i.e., the inhibitor concentrations required to decrease a given enzyme activity by 50%. Tacrine, an effective AChE and BChE inhibitor, and bis-4-nitrophenyl phosphate (BNPP), a selective CES inhibitor, were used as positive controls. The results are shown in Table 1.

As can be seen from Table 1, conjugates **7** generally inhibited both cholinesterases effectively, exhibiting, like tacrine, a higher activity toward BChE. The activities of compounds **7b**,**f**,**i**,**k** obtained here coincided with the previously determined results [71]. Conjugates **7** practically did not inhibit CES, which suggests that potential unwanted drug-drug interactions would be lacking if these compounds were used clinically for AD treatment.

The degree and selectivity of enzyme inhibition was found to be determined by the structure of the synthesized conjugates; namely, the aliphatic ring size of the 4-amino-2,3-polymethylene-quinoline fragment (i.e., the tacrine moiety) and the length of the spacer linking the pharmacophores. The effect of the aliphatic ring size was demonstrated by compounds **7b**, **7f**, **7i**, **7k** with a spacer length *m* = 3 (Table 1). The most active compound toward AChE was conjugate **7f** with an unmodified six-membered tacrine ring (C-6, *n* = 2), whose inhibitory activity was only three times lower than that of tacrine. The conjugate **7i** with a seven-membered tacrine ring (C-7, *n* = 3) exhibited maximum activity toward BChE: it inhibited the enzyme in the nanomolar range with an IC_50_ = 0.044 μM, close to that of tacrine. An increase in the size of the aliphatic ring to 8 (C-8, **7k**) reduced the anti-AChE activity ten-fold and decreased anti-BChE activity six-fold. Thus, the six-membered aliphatic ring of tacrine was optimal for the anti-AChE activity of conjugates **7**, whereas a 7-membered ring was best for their anti-BChE potency.

An increase in the spacer length (*m*) for conjugates with the same ring size in series **7a**–**7e** (C-5, *n* = 1), **7f**–**7h** (C-6, *n* = 2), and **7i**–**7j** (C-7, *n* = 3) led to an increase in the inhibitory activity of compounds toward AChE. The most active among compounds with a five-membered ring were compounds **7c** and **7d** containing four and five methylene groups, respectively. In a series of compounds with a six-membered tacrine aliphatic ring **7f**–**7h** (C-6, *n* = 2), the inhibitory activity toward AChE increased more than 14 times with an increase in the length of the spacer from three to five CH_2_ groups. Compound **7h** exhibited the maximum anti-AChE activity, being five times more potent than tacrine.

The results are consistent with the known data that, in contrast to extremely powerful bis-tacrine inhibitors [78], tacrine-based hybrid compounds linking tacrine with an uncharged aromatic group had anti-AChE activities comparable to that of tacrine [79,80].

In the case of anti-BChE activity, it was not possible to reveal a clear pattern of the dependence of enzyme inhibition on the spacer length of the conjugate. The maximum anti-BChE activity, close to that of tacrine, was found for compounds **7g** and **7h** with a six-membered tacrine ring and a spacer length of four and five CH_2_ groups, respectively, as well as for compounds **7i** and **7j** with a seven-membered tacrine aliphatic ring and a spacer length of three and four CH_2_ groups, respectively.

Regarding inhibitory selectivity, all conjugates **7** were more selective toward BChE, with selectivity factors ranging from 1.9 to 64. Conjugates with a rather short propylene spacer (**7b, 7f, 7i** and **7k**) exhibited maximum selectivity for BChE (S = 17.1–64.0). The most selective was conjugate **7i** with a 7-membered aliphatic ring.

### 2.3. Kinetic Studies of AChE and BChE Inhibition

**Figure 3:**
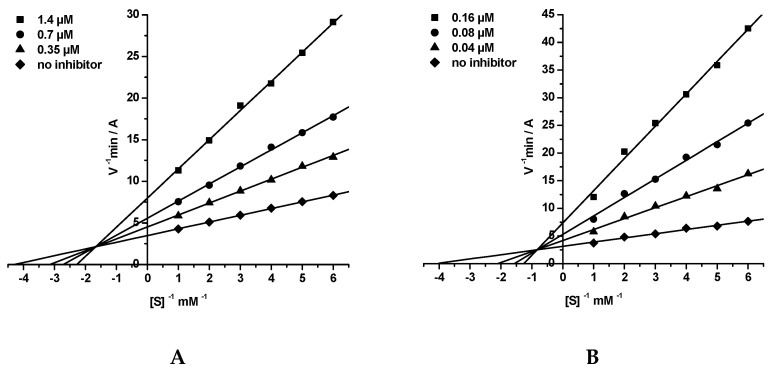
Steady state inhibition of (**A**) AChE and (**B**) BChE by compound **7g**. Lineweaver-Burk double-reciprocal plots of initial velocity and substrate concentrations in the presence of inhibitor (three concentrations) and without inhibitor are presented. The changes in both *K*_m_ and *V*_max_ attest to a mixed type of inhibition.

The mechanism of inhibitory action of conjugates **7** towards AChE and BChE was studied using compound **7g** as an example. The graphical analysis of the kinetic data on AChE (Figure 3A) and BChE (Figure 3B) inhibition by **7g** in the Lineweaver–Burk double-reciprocal plot demonstrates the changes in both *K*_m_ and *V*_max_ that attests to a mixed type of inhibition. The inhibition constants were *K*_i_ = 0.390 ± 0.008 μM (competitive component) and α*K*_i_ = 1.09 ± 0.02 μM (noncompetitive component) for AChE and *K*_i_ = 0.0235 ± 0.0002 μM (competitive component) and α*K*_i_ = 0.117 ± 0.002 μM (noncompetitive component) for BChE.

### 2.4. Molecular Docking Studies

The differential interaction of bulky inhibitors with AChE and BChE is highly dependent on the difference in structure of the active site gorge that leads from the protein surface to the active site [49,50,81,82,83,84]. AChE has a narrow gorge divided by the so-called bottleneck into the PAS and CAS [85,86]. The AChE gorge is lined with aromatic residues that favor binding of positively charged substrates/inhibitors, and the dynamically changing radius of the bottleneck determines the kinetics of binding; in particular, slow-binding inhibition [87,88]. BChE has a wider gorge than AChE without a pronounced narrowing to separate the PAS and CAS, and most of the BChE gorge residues that are aromatic in AChE are replaced in BChE with aliphatic ones [86]. This makes the BChE gorge more permissive to uncharged or even negatively charged substrates and inhibitors [89], and less prone to slow-binding inhibition than is the case with AChE [90,91].

Molecular docking of conjugates **7** into AChE demonstrated that they are dual binding-site inhibitors. This confirms our previous finding [49,71]. The tacrine fragment binds in the CAS and the *p*-tolylsulfonamide moiety binds to the PAS. Comparison of docking results of the compounds to different X-ray structures of human AChE showed that the best binding poses were obtained with the X-ray structure of AChE co-crystallized with donepezil (PDB: 4EY7). This structure has conformational changes of CAS and PAS amino acids necessary to accommodate bulky inhibitors [92].

**Figure 4:**
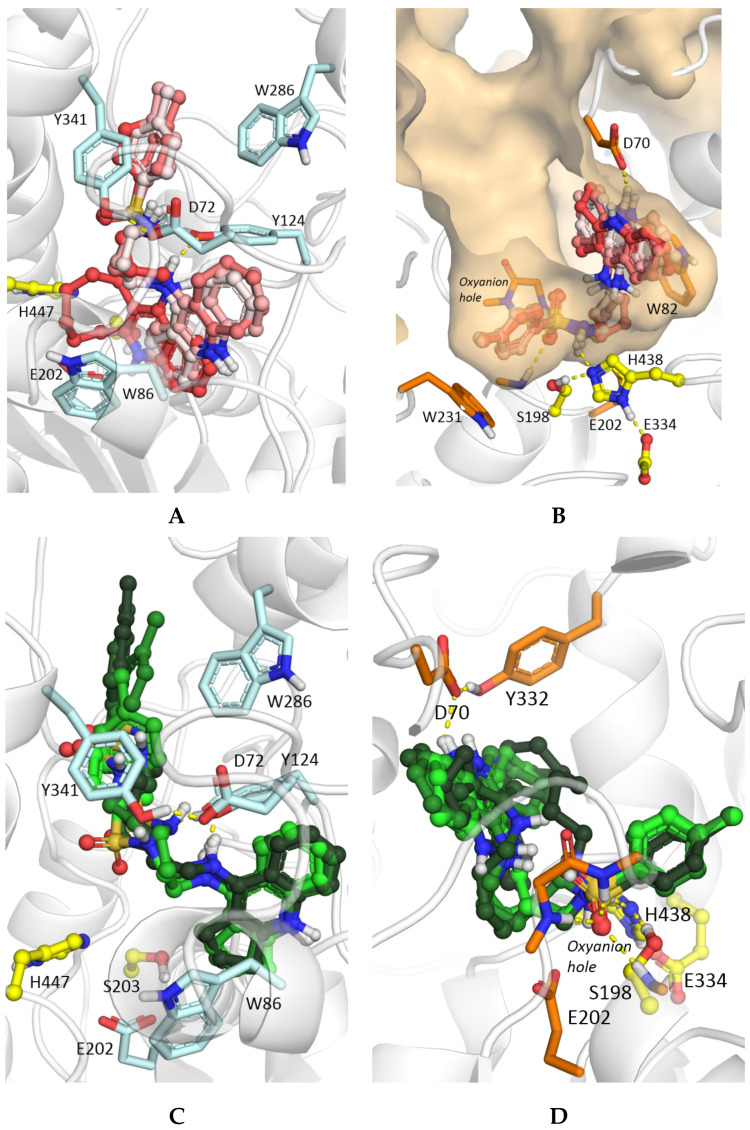
Results of molecular docking of hybrids of 4-amino-2,3-polymethylene-quinoline and *p*-tolylsulfonamide **7** into AChE (carbon atoms are shown in pale blue) and BChE (carbon atoms are shown in orange). The surface shows the gorge leading from the protein surface to the active site with a hollow space accommodating the aliphatic ring of the tacrine fragment of compounds **7**. Compounds **7b**, **7f**, **7i**, **7k** with *m* = 3 spacers and increasing aliphatic ring size (carbon atoms are shown with different shades of red from pale for **7b** (C-5) to bright for **7k** (C-8) inside (**A**) AChE and (**B**) BChE. Compounds **7a–7e** with C-5 aliphatic rings and increasing spacer length (carbon atoms are shown with different shades of green from pale for **7a** (*m* = 2) to dark for **7e** (*m* = 6) inside (**C**) AChE and (**D**) BChE.

Molecular docking of compounds **7b**, **7f**, **7i**, **7k** with the spacer *m* = 3 explains the observed effect of the aliphatic ring size on the inhibitory activity toward AChE. The position of the 4-amino-2,3-polymethylene-quinoline (tacrine) ring inside the AChE active site is similar for compounds **7b**, **7f**, **7i,** but not for compound **7k** with the largest C-8 aliphatic ring (Figure 4A). The increased size of the aliphatic ring does not allow it to fit in the same region below the bottleneck; the displaced C-8 tacrine ring leads to the loss of a hydrogen bond between the protonated amino group of the tacrine fragment and the Tyr124 side chain. This results in the experimentally observed reduced binding affinity.

In the case of binding to BChE, the influence of the aliphatic ring size is not as pronounced as with AChE; all tacrine fragments are located inside the BChE gorge in a uniform way (Figure 4B), with the sulfonamide group binding to the active site and forming hydrogen bonds with catalytic residues and the oxyanion hole. The protonated amino group in the tacrine fragment forms ionic interactions with Asp70 in the PAS. However, for compound **7k** (C-8), the large aliphatic ring size impairs ionic interactions of the positively charged external amino group of the tacrine fragment with the Asp70 anionic side chain. Otherwise, compound **7i** with a 7-membered ring did not have more specific interactions than compounds **7b** and **7f**, but it appeared to fit better than the others in the active site gorge cavity.

As was observed earlier for other dual binding site AChE inhibitors with lengthy spacers [49,50,81,82,83,84], they are located inside the AChE gorge in an elongated conformation whereas they are bent inside the wider BChE gorge. Consequently, an increase in the spacer length for conjugates with the same ring size leads to better occupation of the AChE PAS by the *p*-tolylsulfonamide fragment. Figure 4C shows binding poses of compounds **7a**–**7e** (C-5) as an example, while for compounds **7f**–**7h** (C-6) the same trend was observed. Although for the compounds with a shorter linker (**7a**, **7b**) hydrogen bonding between the sulfo-group and the Tyr124 side chain is possible while no specific interactions of the conjugate molecules in the PAS were observed, more favorable occupancy of the PAS by compounds with a longer linker leads to better propidium displacement and increased inhibitory activity due to hydrophobic interactions. In contrast, the position of the compounds inside the BChE gorge was almost unaffected by spacer length (Figure 4D), in accordance with the weak influence of the spacer length on experimentally measured inhibitory activities.

Thus, the molecular docking results show that the differences in the structure of the active sites of AChE and BChE give rise to different structure-inhibitory activity relationships for inhibition of these enzymes by these hybrid inhibitors.

### 2.5. Displacement of Propidium Iodide from the PAS of EeAChE

Propidium is a selective ligand for the PAS of AChE responsible for the Aβ binding [24,29,30,93], which showed a significant decrease in AChE-induced Aβ aggregation (82% at 100 µM) [26]. This observation has served as the basis for the fluorescent evaluation of competitive propidium displacement from the PAS of AChE as a screening method to assess the potential ability of compounds to bind PAS and thus block the pro-aggregation activity of AChE [28]. Propidium iodide exhibits a fluorescence increase upon binding to AChE. A decrease in propidium fluorescence in the presence of test compounds indicates that they are able to displace propidium and bind to the PAS of AChE, suggesting that they would thereby block the AChE-mediated aggregation of β-amyloid [24,27,29,33]. Here, donepezil was used as a reference compound. Donepezil is a mixed-type AChE inhibitor, for which the ability to block AChE-PAS-induced Aβ aggregation has been demonstrated [26]. The results are presented in Table 1.

It can be seen in Table 1 that the sulfonamide-containing conjugates **7** at a concentration of 20 μM reduced the fluorescence intensity by 9–17% and more effectively displaced propidium from the PAS of AChE compared to the reference compound donepezil. For compounds **7f**, **i**, there were no differences with the results obtained earlier [71]. For all 4-amino-2,3-polymethylene-quinoline–sulfonamide heterodimers with five, six, or seven-membered aliphatic rings, the degree of propidium displacement increased with elongation of the spacer length, which agrees with the results of molecular docking.

Taken together, the results from propidium displacement, kinetics, and molecular docking indicate that the conjugates **7** are dual-site AChE inhibitors occupying both CAS and PAS pockets. Thanks to binding to the PAS of AChE, they can potentially block AChE-induced aggregation of β-amyloid, thus having an additional positive disease modification effect.

### 2.6. Antioxidant Activity

The presence of antioxidant activity in a number of sulfonamide derivatives [66,68,69] prompted us to assess the antioxidant properties of the new hybrid compounds. A study of the antioxidant activity of conjugates **7** in the ABTS test showed that the binding of the ABTS radical by these conjugates is rather weak in comparison with the standard antioxidant, Trolox. TEAC values = 0.06–0.13 (TEAC values are expressed as Trolox equivalents calculated from (A_0_–A_test_)/(A_0_–A_Trolox_) at 20 µM concentrations, where A_0_ is the absorbance of a control lacking any radical scavenger, A_test_ and A_Trolox_ are the absorbances of the remaining ABTS^•+^ in the presence of the test compound or Trolox, respectively).

### 2.7. Predicted ADMET Profiles and PAINS Analysis

The results of the computational estimation of a number of ADMET and physicochemical properties for the compounds **7a**–**k** are shown in Table 2. As can be seen, all of the compounds had high predicted values for intestinal absorption, enabling their oral administration. Thanks to moderate predicted blood-brain barrier permeability (brain concentration is about 10–25% of the plasma concentration), sufficient CNS activity can be expected. Both parameters of the cardiac toxicity risk (p*K_i_* and pIC_50_) for all analyzed compounds (4.7–6.5 log units) are in the lower or medium part of their possible range (3–9 log units). Although these values are acceptable, additional studies and structure optimization are desirable for a few compounds in order to ensure maximal safety. The predicted lipophilicities and aqueous solubilities of the compounds are also good for potential drug compounds. The integral quantitative estimates of drug-likeness (QED) are above 0.3 for all the compounds and above 0.5 for most of them. The Pan Assay INterference compoundS (PAINS) filter check for the compounds listed in Table 2 did not identify any structural alerts. Thus, the predicted ADMET, physicochemical, and PAINS properties of the compounds **7a**–**k** are quite acceptable for potential lead compounds at the early drug development stages.

## 3. Materials and Methods

### 3.1. Chemistry

All solvents, chemicals, and reagents were obtained commercially and used without purification. ^1^H-NMR (200 MHz) spectra were recorded on a DPX-200 NMR spectrometer (Bruker, Karlsruhe, Germany) using tetramethylsilane as an internal standard. Chemical shifts, δ, are given in parts per million (ppm), and spin multiplicities are given as s (singlet), br s (broad singlet), d (doublet), t (triplet), q (quartet) or m (multiplet). Coupling constants, *J*, are expressed in hertz (Hz). Melting points were recorded on a SMP10 Melting Point Apparatus (Stuart, Staffordshire, UK) and are uncorrected. Yields refer to isolated pure products and were not maximized. CHN analysis was performed on the ER-20 analyzer (Carlo-Erba, Val-de-Reuil, France). All compounds exhibited analytical and spectroscopic data that strongly agreed with their expected structures.

### 3.2. Synthesis of Compounds

The synthesis and characteristics of compounds are described below. The original NMR spectra are presented in the Supplementary Materials.

#### General Procedure for the Preparation of Derivatives **7a**–**k**

A solution of tosyl chloride (190.65 mg, 1 mmol) in methylene chloride (5 mL) was added to a solution of NEt_3_ (2 mmol) and aminoquinoline **5** (1 mmol) in methylene chloride (4 mL). The reaction mixture was stirred for 1 h at room temperature, diluted with 10 mL of water, and extracted with ethyl acetate (3 × 10 mL). The combined extracts were washed with brine, dried over anhydrous Na_2_SO_4,_ and evaporated under vacuum to give the required crude product that was recrystallized from isopropanol.

*N-(2-(2,3-Dihydro-1H-cyclopenta[b]quinolin-9-ylamino)-ethyl)-4-methyl-benzenesulfonamide* (**7a**). White solid; Yield 75%, m.p. 245–247 °C. ^1^H-NMR (DMSO-d_6_) δ: 2.17 (m, 2H, CH_2_), 2.33 (s, 3H, CH_3_), 2.80 (m, 2H, CH_2_), 3.19 (m, 2H, CH_2_), 3.83 (m, 4H, 2 × CH_2_), 7.17 (d, 2H, *J* = 8.4 Hz, H_ar_), 7.53 (t, 1H, *J* = 7.2 Hz, H_ar_), 7.61 (d, 2H, *J* = 8.4 Hz, H_ar_), 7.76 (t, 2H, *J* = 7.3 Hz, H_ar_), 7.90 (m, 2H, NH, H_ar_), 8.43 (m, 2H, NH, H_ar_). Anal. Calcd for C_21_H_23_N_3_O_2_S: C, 66.12; H, 6.08; N, 11.01. Found: C, 66.18; H, 6.21; N, 11.12.

*N-(3-(2,3-Dihydro-1H-cyclopenta[b]quinolin-9-ylamino)-propyl)-4-methyl-benzenesulfonamide* (**7b**). White solid; Yield 72%, m.p. 213–215 °C. ^1^H-NMR (DMSO-d_6_ + CDCl_3_) δ: 1.77 (m, 2H, CH_2_), 2.12 (m, 2H, CH_2_), 2.29 (s, 3H, CH_3_), 2.83 (m, 2H, CH_2_), 3.19 (m, 4H, 2 × CH_2_), 3.75 (q, 2H, *J* = 6.1 Hz, CH_2_), 7.03 (d, 2H, *J* = 7.8 Hz, H_ar_), 7.30 (m, 2H, NH, H_ar_), 7.48 (m, 3H, NH, H_ar_), 7.80 (d, 1H, *J* = 8.2 Hz, H_ar_), 8.23 (m, 1H, NH), 8.25 (d, 1H, *J* = 8.6 Hz, H_ar_). Anal. Calcd for C_22_H_25_N_3_O_2_S: C, 66.81, H, 6.37, N, 10.62. Found: C, 66.61, H, 6.47, N, 10.44.

*N-(4-(2,3-Dihydro-1H-cyclopenta[b]quinolin-9-ylamino)-butyl)-4-methyl-benzenesulfonamide* (**7c**). White solid; Yield 65%, m.p. 215–217 °C. ^1^H-NMR (DMSO-d_6_) δ: 1.51 (m, 2H, CH_2_), 1.68 (m, 2H, CH_2_), 2.20 (m, 2H, CH_2_), 2.40 (s, 3H, CH_3_), 2.75 (q, 2H, *J* = 6.2 Hz, CH_2_), 3.26 (m, 4H, 2 × CH_2_), 3.70 (q, 2H, *J* = 6.2 Hz, CH_2_), 7.28 (d, 2H, *J* = 8.2 Hz, H_ar_), 7.51 (m, 2H, NH, H_ar_), 7.63 (d, 2H, *J* = 8.4 Hz, H_ar_), 7.76 (t, 1H, *J* = 7.4 Hz, H_ar_), 7.95 (d, 1H, *J* = 8.2 Hz, H_ar_), 8.54 (d, 1H, *J* = 8.2 Hz, H_ar_), 8.80 (t, 1H, *J* = 6.2 Hz, NH). Anal. Calcd for C_23_H_27_N_3_O_2_S: C, 67.45, H, 6.65, N, 10.26. Found: C, 67.57, H, 6.61, N, 10.41.

*N-(5-(2,3-Dihydro-1H-cyclopenta[b]quinolin-9-ylamino)-pentyl)-4-methyl-benzenesulfonamide* (**7d**). White solid; Yield 76%, m.p. 173–175 °C. ^1^H-NMR (DMSO-d_6_) δ: 1.57 (m, 6H, 3 × CH_2_), 2.19 (m, 2H, CH_2_), 2.38 (s, 3H, CH_3_), 2.75 (q, 2H, *J* = 6.3 Hz, CH_2_), 3.26 (m, 4H, 2 × CH_2_), 3.67 (q, 2H, *J* = 6.3 Hz, CH_2_), 7.25 (d, 2H, *J* = 8.2 Hz, H_ar_), 7.47 (m, 2H, NH, H_ar_), 7.62 (d, 2H, *J* = 8.2 Hz, H_ar_), 7.76 (t, 1H, *J* = 7.3 Hz, H_ar_), 7.93 (d, 1H, *J* = 8.2 Hz, H_ar_), 8.49 (d, 1H, *J* = 8.2 Hz, H_ar_), 8.76 (t, 1H, *J* = 6.2 Hz, NH). Anal. Calcd for C_24_H_29_N_3_O_2_S: C, 68.05, H, 6.90, N, 9.92. Found: C, 68.29, H, 6.77, N, 9.78.

*N-(6-(2,3-Dihydro-1H-cyclopenta[b]quinolin-9-ylamino)-hexyl)-4-methyl-benzenesulfonamide* (**7e**). White solid; Yield 73%, m.p. 230–232 °C. ^1^H-NMR (DMSO-d_6_) δ: 1.57 (m, 6H, 4 × CH_2_), 2.20 (m, 2H, CH_2_), 2.39 (s, 3H, CH_3_), 2.78 (q, 2H, *J* = 6.2 Hz, CH_2_), 3.20 (m, 2H, CH_2_), 3.30 (m, 2H, CH_2_), 3.72 (q, 2H, *J* = 6.2 Hz, CH_2_), 7.23 (d, 2H, *J* = 8.2 Hz, H_ar_), 7.44 (m, 2H, NH, H_ar_), 7.58 (d, 2H, *J* = 8.2 Hz, H_ar_), 7.73 (t, 1H, *J* = 7.3 Hz, H_ar_), 8.00 (d, 1H, *J* = 8.2 Hz, H_ar_), 8.33 (d, 1H, *J* = 8.2 Hz, H_ar_), 8.73 (t, 1H, *J* = 6.2 Hz, NH). Anal. Calcd for C_25_H_31_N_3_O_2_S: C, 68.62, H, 7.14, N, 9.60. Found: C, 68.90, H, 7.27, N, 9.44.

*4-Methyl-N-(3-(1,2,3,4-tetrahydro-acridin-9-ylamino)-propyl)-benzenesulfonamide* (**7f**). White solid; Yield 68%, m.p. 222–224 °C. ^1^H-NMR (DMSO-d_6_) δ: 1.88 (m, 6H, 3 × CH_2_), 2.38 (s, 3H, CH_3_), 2.67 (m, 2H, CH_2_), 2.80 (q, 2H, *J* = 6.2 Hz, CH_2_), 3.07 (m, 2H, CH_2_), 3.90 (q, 2H, *J* = 5.6 Hz, CH_2_), 7.28 (d, 2H, *J* = 8.2 Hz, H_ar_), 7.51 (t, 1H, *J* = 7.7 Hz, H_ar_), 7.63 (d, 2H, *J* = 8.2 Hz, H_ar_), 7.79 (m, 3H, 2 × NH, H_ar_), 8.08 (d, 1H, *J* = 8.5 Hz, H_ar_), 8.39 (d, 1H, *J* = 8.5 Hz, H_ar_). Anal. Calcd for C_23_H_27_N_3_O_2_S: C, 67.45, H, 6.65, N, 10.26. Found: C, 67.66, H, 6.70, N, 10.22.

*4-Methyl-N-(4-(1,2,3,4-tetrahydro-acridin-9-ylamino)-butyl)-benzenesulfonamide* (**7g**). White solid; Yield 71%, m.p. 165–167 °C. ^1^H-NMR (CDCl_3_) δ: ^1^H-NMR (CDCl_3_) δ: 1.69 (m, 4H, 2 × CH_2_), 1.84 (m, 4H, 2 × CH_2_), 2.38 (s, 3H, CH_3_), 2.62 (m, 2H, CH_2_), 2.95 (m, 2H, CH_2_), 3.07 (m, 2H, CH_2_), 3.58 (q, 2H, *J* = 6.6 Hz, CH_2_), 4.77 (m, 1H, NH), 5.45 (m, 1H, NH), 7.23 (d, 2H, *J* = 7.8 Hz, H_ar_), 7.31 (m, 1H, H_ar_), 7.53 (m, 1H, H_ar_), 7.74 (d, 2H, *J* = 8.4 Hz, H_ar_), 7.95 (d, 1H, *J* = 8.8 Hz, H_ar_), 8.02 (d, 1H, *J* = 8.2 Hz, H_ar_). Anal. Calcd for C_24_H_29_N_3_O_2_S: C, 68.05, H, 6.90, N, 9.92. Found: C, 68.31, H, 6.72, N, 9.81.

*4-Methyl-N-(5-(1,2,3,4-tetrahydro-acridin-9-ylamino)-pentyl)-benzenesulfonamide* (**7h**). White solid; Yield 73%, m.p. 235–237 °C. ^1^H-NMR (CDCl_3_) δ: 1.69 (m, 6H, 3 × CH_2_), 1.97 (m, 4H, 2 × CH_2_), 2.36 (s, 3H, CH_3_), 2.63 (m, 2H, CH_2_), 2.94 (m, 2H, CH_2_), 3.08 (m, 2H, CH_2_), 3.56 (q, 2H, *J* = 6.7 Hz, CH_2_), 4.75 (m, 1H, NH), 5.45 (m, 1H, NH), 7.24 (d, 2H, *J* = 7.8 Hz, H_ar_), 7.28 (m, 1H, H_ar_), 7.53 (t, 1H, *J* = 7.1 Hz, H_ar_), 7.74 (d, 2H, *J* = 8.5 Hz, H_ar_), 7.96 (d, 1H, *J* = 8.8 Hz, H_ar_), 8.04 (d, 1H, *J* = 8.3 Hz, H_ar_). Anal. Calcd for C_25_H_31_N_3_O_2_S: C, 68.62, H, 7.14, N, 9.60. Found: C, 68.76, H, 7.32, N, 9.52.

*4-Methyl-N-(3-(7,8,9,10-tetrahydro-6H-cyclohepta[b]quinolin-11-ylamino)-propyl)-benzenesulfonamide* (**7i**). White solid; Yield 65%, m.p. 213–215 °C. ^1^H-NMR (DMSO-d_6_) δ: 1.64 (m, 2H, CH_2_), 1.85 (m, 6H, 3 × CH_2_), 2.35 (s, 3H, CH_3_), 2.76 (q, 2H, *J* = 6.1 Hz, CH_2_), 2.88 (m, 2H, CH_2_), 3.23 (m, 2H, CH_2_), 3.72 (m, 2H, CH_2_), 7.31 (d, 2H, *J* = 8.3 Hz, CH_2_), 7.58 (d, 2H, *J* = 8.3 Hz, H_ar_), 7.71 (m, 3H, 2 × NH, H_ar_), 7.88 (t, 1H, *J* = 8.1 Hz, H_ar_), 8.06 (d, 1H, *J* = 8.3 Hz, H_ar_), 8.42 (d, 1H, *J* = 8.3 Hz, H_ar_). Anal. Calcd for C_24_H_29_N_3_O_2_S: C, 68.05, H, 6.90, N, 9.92. Found: C, 68.30, H, 6.72, N, 9.91.

*4-Methyl-N-(4-(7,8,9,10-tetrahydro-6H-cyclohepta[b]quinolin-11-ylamino)-butyl)-benzenesulfonamide* (**7j**). White solid; Yield 66%, m.p. 235–237 °C. ^1^H-NMR (CDCl_3_) δ: 1.60 (m, 4H, 2 × CH_2_), 1.81 (m, 6H, 3 × CH_2_), 2.39 (s, 3H, CH_3_), 2.63 (m, 2H, CH_2_), 2.95 (m, 2H, CH_2_), 3.15 (m, 2H, CH_2_), 3.92 (m, 2H, CH_2_), 7.27 (m, 2H, H_ar_), 7.57 (t, 1H, *J* = 7.6 Hz, H_ar_), 7.78 (d, 2H, *J* = 8.3 Hz, H_ar_), 7.80 (m, 1H, H_ar_), 8.23 (d, 1H, *J* = 8.3 Hz, H_ar_), 8.30 (d, 1H, *J* = 8.3 Hz, H_ar_). Anal. Calcd for C_25_H_31_N_3_O_2_S: C, 68.62, H, 7.14, N, 9.60. Found: C, 68.66, H, 7.01, N, 9.76.

*N-(3-(6,7,8,9,10,11-Hexahydro-cycloocta[b]quinolin-12-ylamino)-propyl)-4-methyl-benzenesulfonamide* (**7k**). White solid; Yield 65%, m.p. 210–212 °C. ^1^H-NMR (DMSO-d_6_) δ: 1.36 (m, 2H, CH_2_), 1.53 (m, 2H, CH_2_), 1.71 (m, 2H, CH_2_), 1.94 (m, 4H, 2 × CH_2_), 2.41 (s, 3H, CH_3_), 2.84 (q, 2H, *J* = 6.1 Hz, CH_2_), 2.95 (m, 2H, CH_2_), 3.25 (m, 2H, CH_2_), 3.92 (m, 2H, CH_2_), 7.27 (d, 2H, *J* = 8.2 Hz, H_ar_), 7.52 (m, 1H, H_ar_), 7.66 (d, 2H, *J* = 8.4 Hz, H_ar_), 7.78 (m, 3H, 2 × NH, H_ar_), 8.33 (m, 2H, H_ar_). Anal. Calcd for C_25_H_31_N_3_O_2_S: C, 68.62, H, 7.14, N, 9.60. Found: C, 68.43, H, 7.07, N, 9.84.

### 3.3. Biological Assays

#### 3.3.1. Enzymatic Assays

- In vitro AChE, BChE and CES Inhibition

All experiments were carried out in accordance with the standard protocols approved by IPAC RAS. Human erythrocyte AChE, equine serum BChE, porcine liver CES, acetylthiocholine iodide (ATCh), butyrylthiocholine iodide (BTCh), 5,5′-dithio-bis-(2-nitrobenzoic acid) (DTNB), and 4-nitrophenol acetate (4-NPA) were purchased from Sigma-Aldrich (Saint Louis, MO, USA).

AChE and BChE activities were measured by the colorimetric method of Ellman et al. [94]. The assay solution consisted of 0.1 M K/Na phosphate buffer pH 7.5, 25 °C, 0.33 mM DTNB, 0.02 unit/mL AChE or BChE, and 1 mM substrate (ATCh or BTCh, respectively). Reagent blanks consisted of reaction mixtures without substrates.

The activity of CES was determined spectrophotometrically at 405 nm to monitor the release of 4-nitrophenol [95] in 0.1 M K/Na phosphate buffer pH 8.0, 25 °C. Final enzyme and substrate (4-nitrophenyl acetate) concentrations were 0.02 unit/mL and 1 mM, respectively. Assays were carried out with a blank containing all constituents except porcine CES to assess non-enzymatic hydrolysis.

Test compounds were dissolved in DMSO; reaction mixtures contained 2% (*v/v*) of the solvent, a concentration shown not to affect the activity of the enzymes on its own (data not shown). Tacrine, donepezil, and bis-4-nitrophenyl phosphate (BNPP), a selective CES inhibitor, were used as positive controls.

An initial evaluation of inhibitory activity of compounds was carried out by determination of the AChE, BChE and CES inhibition at a compound concentration of 20 µM. For this, a sample of the corresponding enzyme was incubated with the test compound for 5 min at 25 °C for temperature equilibration; then the enzyme residual activity was determined. Each experiment was performed in triplicate.

For the most active compounds, the IC_50_ values (the concentration of inhibitor required to decrease the enzyme activity by 50%) were determined. Eight different concentrations of the test compounds in the range 10^−12^–10^−4^ M were selected in order to obtain inhibition of esterase activity between 20% and 80%. The test compounds were added to the assay solution and incubated with the enzyme at 25 °C for 5 min (for temperature equilibration) followed by the addition of substrate. A parallel control was made for the assay solution with no inhibitor.

Measurements were performed in the FLUOStar OPTIMA microplate spectrophotometer (BMG Labtech, Ortenberg, Germany). Each experiment for the IC_50_ assay was performed in triplicate. The results were expressed as the mean ± SEM. The reaction rates in the presence and absence of inhibitor were compared, and the percent residual enzyme activity due to the presence of test compounds was calculated. IC_50_ values were determined graphically from inhibition curves (log inhibitor concentration vs. percent residual enzyme activity) using Origin 6.1 software (OriginLab, Northampton, MA, USA).

- Kinetic Study of AChE and BChE Inhibition. Determination of Steady-State Inhibition Constants

Mechanisms of AChE and BChE inhibition were assessed via a thorough analysis of enzyme kinetics. Residual activity was measured following 5 min incubation at 25 °C (for temperature equilibration) with three increasing concentrations of inhibitor and six decreasing substrate concentrations. Substrate was added immediately after the 5 min incubation with inhibitor and the rates of absorption were monitored at 412 nm using the FLUOStar OPTIMA (BMG Labtech) microplate spectrophotometer. Inhibition constants *K_i_* (competitive component) and *αK_i_* (noncompetitive component) were determined by linear regression of 1/V versus 1/[S] double-reciprocal (Lineweaver-Burk) plots using Origin 6.1 software. Data were expressed as mean ± SEM (*n* = 3 experiments).

#### 3.3.2. Propidium Displacement Studies

The ability of the test compounds to competitively displace propidium, a selective ligand of the PAS of AChE, was evaluated by the fluorescence method [93,96]. *Ee*AChE was used owing to its high degree of purification, high activity, and lower cost than human AChE (hAChE). In addition, we performed 3D alignment of the crystal structures of *Ee*AChE (PDB: 1C2O) and hAChE (PDB: 4EY7) using YASARA-Structure 18.4.24 for Windows, which showed that the two structures were essentially congruent with an RMSD of 0.623 Å over 527 aligned residues and 88.6% sequence identity. The fluorescence intensity of propidium iodide bound with AChE increases several times; decreasing fluorescence intensity of the bound propidium in the presence of the test compounds shows their ability to bind to the PAS of AChE [24,27].

To determine the degree of displacement (% displacement) of propidium from the PAS of AChE, *Ee*AChE (final concentration, 7 μM) was incubated with the test compound at a concentration of 20 μM in 1 mM Tris-HCl buffer pH 8.0, 25 °C, for 15 min. Then, propidium iodide solution (final concentration, 8 μM) was added, the samples were incubated for 15 min and the fluorescence spectrum (530 nm (excitation) and 600 nm (emission)) was taken. Donepezil and tacrine were used as reference compounds. The blank contained propidium iodide of the same concentration in 1 mM Tris-HCl buffer pH 8.0. The measurements were carried out in triplicate on the FLUOStar Optima microplate reader (BMG Labtech), and the results were calculated by the following formula:

% Displacement = 100 − (IF_AChE+ Propidium + inhibitor_/IF_AChE + Propidium_) × 100 (1)

where IF_AChE + Propidium_ is the fluorescence intensity of the propidium associated with AChE in the absence of the test compound (taken as 100%), and IF_AChE + Propidium + inhibitor_ is the fluorescence intensity of the propidium associated with AChE in the presence of the test compound.

#### 3.3.3. Antioxidant Activity: ABTS Radical Cation Scavenging Activity Assay

Radical scavenging activity of the compounds was assessed using the ABTS radical decolorization assay [97] with some modifications [50]. ABTS was purchased from Tokyo Chemical Industry Co. (Tokyo, Japan), potassium persulfate (dipotassium peroxodisulfate), Trolox^®^ (6-hydroxy-2,5,7,8-tetramethychroman-2-carboxylic acid), and HPLC grade ethanol were obtained from Sigma-Aldrich. Aqueous solutions were prepared using deionized water.

The solution of cation radical ABTS^•+^ was produced by incubation of ABTS with potassium persulfate in deionized water for 12–16 h at room temperature in the dark. Radical scavenging capacity of the compounds was analyzed by mixing 10 μL of compound with 240 μL of ABTS^•+^ working solution in ethanol (100 μM final concentration). After 1 h of incubation, the reduction in absorbance was measured spectrophotometrically at 734 nm using the xMark microplate UV/VIS microplate spectrophotometer (Bio-Rad, Hercules, CA, USA). Ethanol blanks were run in each assay. Values were obtained from three replicates of each sample and three independent experiments. Trolox was used as the antioxidant standard.

The antioxidant activity is reported as Trolox equivalent antioxidant capacity (TEAC values) by comparing (A_0_ − A_test_) of the test antioxidant with (A_0_ − A_Trolox_) of the Trolox standard at a concentration of 20 μM after a reaction time of 1 h. A_0_ is the absorbance of a control lacking any radical scavenger and A_test/Trolox_ is the absorbance of the remaining ABTS^•+^ in the presence of the test compound or Trolox, respectively:

TEAC = (A_0_ − A_test_)/(A_0_ − A_Trolox_) (2)

### 3.4. Molecular Modeling Studies

X-ray structures of human AChE in *apo*-form and co-crystallized with different ligands (PDB: 4EY4–4EY7) [98] and an optimized X-ray structure of human BChE (PDB: 1P0I) [99,100] were used for molecular docking. Ligand structures were optimized using a DFT quantum chemistry method (B3LYP/6-31G*, GAMESS-US [101] software). Partial atomic charges on ligand atoms were assigned from QM data according to the Löwdin scheme [102]. Molecular docking was performed with AutoDock 4.2.6 software [103]. The grid box for docking included the entire active site gorge of AChE (22.5 Å × 22.5 Å × 22.5 Å grid box dimensions) and BChE (15 Å × 20.25 Å × 18 Å grid box dimensions) with a grid spacing of 0.375 Å. The main Lamarckian Genetic Algorithm (LGA) [104] parameters were 256 runs, 25 × 10^6^ evaluations, 27 × 10^4^ generations, and a population size of 3000. Figures were prepared with PyMOL (www.pymol.org).

### 3.5. Prediction of ADMET Profiles and PAINS Analysis

Human intestinal absorption (HIA) [105], blood-brain barrier permeability (LogBB) [106], and hERG-mediated cardiac toxicity risk (channel affinity p*K_i_* and inhibitory activity pIC_50_) [107] were estimated using the integrated online service for ADMET properties prediction (ADMET Prediction Service) [108]. It implements the predictive QSAR models based on accurate and representative training sets, fragmental descriptors, and artificial neural networks. The lipophilicity (LogPow) and aqueous solubility (pS) were estimated by the ALogPS 3.0 neural network model implemented in the OCHEM platform [109]. The quantitative estimate of drug-likeness (QED) values [110] were calculated and the Pan Assay INterference compoundS (PAINS) alerts were checked using the RDKit version 2019.03.4 software [111].

### 3.6. Statistical Analyses

Results are presented as mean ± SEM calculated using GraphPad Prism version 6.05 (San Diego, CA, USA) for Windows. Plots, linear regressions, and IC_50_ values were determined using Origin 6.1 (Northampton, MA, USA) for Windows.

## 4. Conclusions

New hybrids of 4-amino-2,3-polymethylene-quinoline and *p*-tolylsulfonamide with different aliphatic ring sizes and alkylene spacers of increasing length were synthesized and their structures established. All synthesized compounds exhibited high dual anticholinesterase activity and greater selectivity toward BChE. They possessed low anti-CES activity, suggesting the absence of potential unwanted drug-drug interactions in prospective clinical use.

Kinetics studies were consistent with mixed-type reversible inhibition. Molecular docking into AChE and BChE provided detailed insights into the binding modes demonstrating dual-site binding of the conjugates in AChE and clarifying differences in the structure-activity relationships for AChE and BChE inhibition.

The conjugates had the ability to bind to the AChE PAS and displace propidium, indicating their potential to block AChE-induced β-amyloid aggregation, thereby potentially exerting disease-modifying capability. All compounds demonstrated low antioxidant activity.

Computational ADMET profiles predicted that all the conjugates should have good intestinal absorption, medium blood-brain barrier permeability, and medium cardiac toxicity risks. Moreover, the PAINS filter check for the compounds did not identify any alerts.

Overall, the developed hybrid compounds demonstrated potent dual anticholinesterase activity along with the predicted propensity to block AChE-induced aggregation of β-amyloid. Thus, they have the potential for treating symptoms as well as exerting disease-modifying effects and show promise for further development and optimization as multitarget anti-AD agents. In particular, the lead compound 4-methyl-N-(5-(1,2,3,4-tetrahydro-acridin-9-ylamino)-pentyl)benzenesulfonamide (**7h**), with an unmodified six-membered tacrine ring and *m* = 5, exhibited maximum anti-AChE activity with IC_50_ = 0.131 ± 0.01 µM (five times more potent than tacrine) and high anti-BChE activity. It is a mixed-type AChE inhibitor with the highest percentage of propidium displacement, very good intestinal absorption and medium predicted blood-brain barrier permeability.

## Figures and Tables

**Table 1:** Esterase profile of conjugates **7** and their ability to displace propidium.

Compound			Inhibitory Activity Against AChE, BChE and CES and Inhibitor Selectivity				Displacement of Propidium from the *Ee*AChE PAS, (%) ^1^
N	*n*	*m*	Human Erythrocyte AChE, IC_50_ (µM)	Equine Serum BChE, IC_50_ (µM)	Porcine Liver CES, (%)^1^	Selectivity, S = IC_50_ AChE/IC_50_ BChE	
**7a**	1	2	9.03 ± 0.64	0.924 ± 0.031	11.9 ± 1.5	9.8	9.2 ± 1.0
**7b**	1	3	7.76 ± 0.61	0.327 ± 0.004	8.1 ± 0.6	23.7	12.3 ± 1.1
**7c**	1	4	2.08 ± 0.08	0.578 ± 0.025	9.0 ± 0.1	3.6	14.9 ± 1.2
**7d**	1	5	1.97 ± 0.05	0.459 ± 0.044	29.6 ± 1.2	4.3	15.1 ± 1.4
**7e**	1	6	4.00 ± 0.09	0.209 ± 0.008	18.6 ± 0.2	1.9	15.9 ± 1.3
**7f**	2	3	1.88 ± 0.03	0.110 ± 0.005	2.0 ± 0.8	17.1	9.8 ± 0.8
**7g**	2	4	0.668 ± 0.17	0.0617 ± 0.0003	17.1 ± 1.4	10.8	14.8 ± 1.3
**7h**	2	5	0.131 ± 0.01	0.0680 ± 0.0014	6.7 ± 0.8	1.9	17.5 ± 1.5
**7i**	3	3	2.76 ± 0.04	0.0431 ± 0.0011	1.9 ± 0.9	64.0	13.9 ± 1.3
**7j**	3	4	1.16 ± 0.03	0.0788 ± 0.006	10.3 ± 0.8	14.7	16.1 ± 1.4
**7k**	4	3	11.1 ± 0.2	0.461 ± 0.007	5.2 ± 1.5	24.1	15.9 ± 1.7
**Tacrine**			0.601 ± 0.047	0.0295 ± 0.0020	n.a.	20.4	4.4 ± 0.6
**Donepezil**			0.040 ± 0.004	19.2 ± 3.0	n.a.	0.002	10.1 ± 0.6
**BNPP**			n.a.	n.a.	1.80 ± 0.11	n.d.	n.d.

^1^ compound concentration 20 µM. n.a.—not active. n.d.—not determined. Data are presented as means ± SEM, *n* = 3.

**Table 2:** Predicted ADMET and physicochemical profiles of conjugates **7**.

Compound			LogBB	HIA%	hERG, *pK_i_*	hERG, pIC_50_	LogPow	pS	QED
N	*n*	*m*							
**7a**	1	2	−0.94	97	4.67	5.54	2.89	3.60	0.64
**7b**	1	3	−0.86	100	4.88	5.76	3.21	3.73	0.60
**7c**	1	4	−0.78	100	4.96	6.14	3.62	4.02	0.55
**7d**	1	5	−0.70	100	5.25	6.18	4.04	4.34	0.49
**7e**	1	6	−0.63	100	5.03	6.35	4.47	4.68	0.44
**7f**	2	3	−0.78	100	4.92	5.64	3.71	4.09	0.57
**7g**	2	4	−0.70	100	5.25	6.02	4.09	4.38	0.52
**7h**	2	5	−0.63	100	5.30	6.37	4.49	4.70	0.46
**7i**	3	3	−0.70	100	4.93	5.75	4.11	4.40	0.43
**7j**	3	4	−0.63	100	5.01	6.46	4.48	4.69	0.38
**7k**	4	3	−0.63	100	5.04	5.91	4.55	4.75	0.51

Note: LogBB—blood-brain barrier permeability, HIA—human intestinal absorption [%], hERG *pKi*–hERG potassium channel affinity [−log(M)], hERG pIC_50_–hERG potassium channel inhibitory activity [−log(M)], LogPow—octanol-water partition coefficient, pS—aqueous solubility [−log(M)], QED—quantitative estimate of drug-likeness.

## Supplementary Materials

The following are available online: Figures S1–S11: NMR spectra for **7a**–**k**.
