# Supplementary material for: New Hybrids of 4-Amino-2,3-polymethylene-quinoline and p-Tolylsulfonamide as Dual Inhibitors of Acetyl- and Butyrylcholinesterase and Potential Multifunctional Agents for Alzheimer’s Disease Treatment
Source: Molecules. 2020 Aug 27;25(17):3915. doi: 10.3390/molecules25173915 (PMC7504258; doi:10.3390/molecules25173915)
Supplement: Supplementary file 1 [file molecules-25-03915-s001.zip › Suppl_Title.pdf]

New Hybrids of 4-Amino-2,3-polymethylene-quinoline and *p*-Tolylsulfonamide as Dual Inhibitors of Acetyl- and Butyrylcholinesterase and Potential Multifunctional Agents for Alzheimer's Disease Treatment

Galina F. Makhaeva [1], Nadezhda V. Kovaleva [1], Natalia P. Boltneva [1], Sofya V. Lushchekina [1,2], Tatiana Yu. Astakhova [2], Elena V. Rudakova [1], Alexey N. Proshin [1], Igor V. Serkov [1], Eugene V. Radchenko [1,3], Vladimir A. Palyulin [1,3], Sergey O. Bachurin [1] and Rudy J. Richardson [4,5,6,*]

1 Institute of Physiologically Active Compounds, Russian Academy of Sciences, 142432 Chernogolovka, Russia; gmakh@ipac.ac.ru (G.F.M.); kovalevanv@ipac.ac.ru (N.V.K.); boltneva@ipac.ac.ru (N.P.B); sofya.lushchekina@gmail.com (S.V.L.); rudakova@ipac.ac.ru (E.V.R.); proshin@ipac.ac.ru (A.N.P.); serkoviv@mail.ru (I.V.S.); genie@qsar.chem.msu.ru (E.V.R.); vap@qsar.chem.msu.ru (V.A.P.); bachurin@ipac.ac.ru (S.O.B.)

2 Emanuel Institute of Biochemical Physics, Russian Academy of Sciences, 119334 Moscow, Russia; astakhova1967.t@yandex.ru

3 Department of Chemistry, Lomonosov Moscow State University, 119991 Moscow, Russia

4 Department of Environmental Health Sciences, University of Michigan, Ann Arbor, MI 48109, USA

5 Department of Neurology, University of Michigan, Ann Arbor, MI 48109, USA

6 Center of Computational Medicine and Bioinformatics, University of Michigan, Ann Arbor, MI 48109, USA

* Correspondence: rjrich@umich.edu; Tel.: +1-734-936-0769

Table of contents

Contents

Figure S1: NMR spectra for **7a**

Figure S2: NMR spectra for **7b**

Figure S3: NMR spectra for **7c**

Figure S4: NMR spectra for **7d**

Figure S5: NMR spectra for **7e**

Figure S6: NMR spectra for **7f**

Figure S7: NMR spectra for **7g**

Figure S8: NMR spectra for **7h**

Figure S9: NMR spectra for **7i**

Figure S10: NMR spectra for **7j**

Figure S11: NMR spectra for **7k**
