# Supplementary figures and images for: New Hybrids of 4-Amino-2,3-polymethylene-quinoline and p-Tolylsulfonamide as Dual Inhibitors of Acetyl- and Butyrylcholinesterase and Potential Multifunctional Agents for Alzheimer’s Disease Treatment
Source: Molecules. 2020 Aug 27;25(17):3915. doi: 10.3390/molecules25173915 (PMC7504258; doi:10.3390/molecules25173915)

**7a**

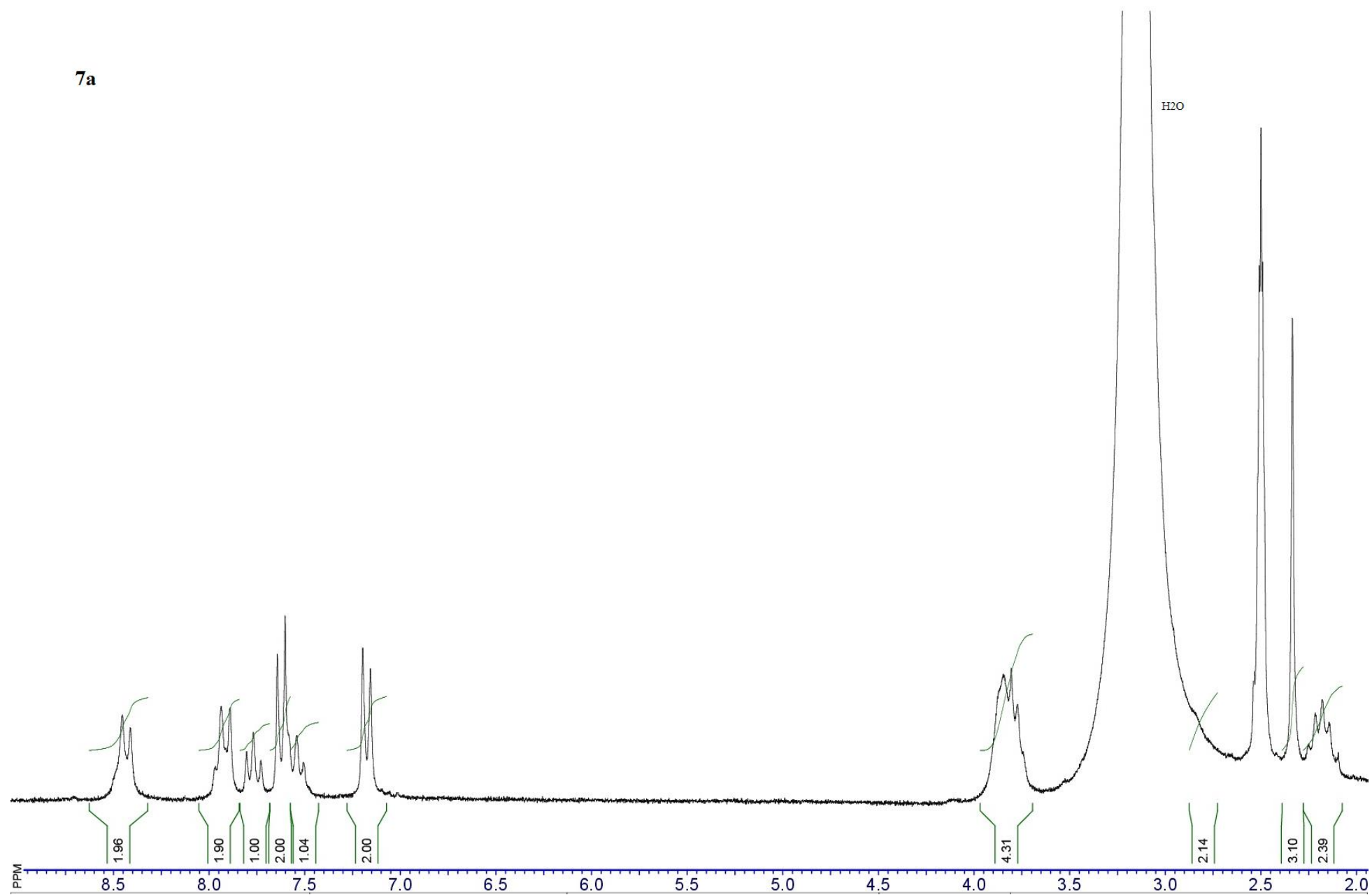

7b

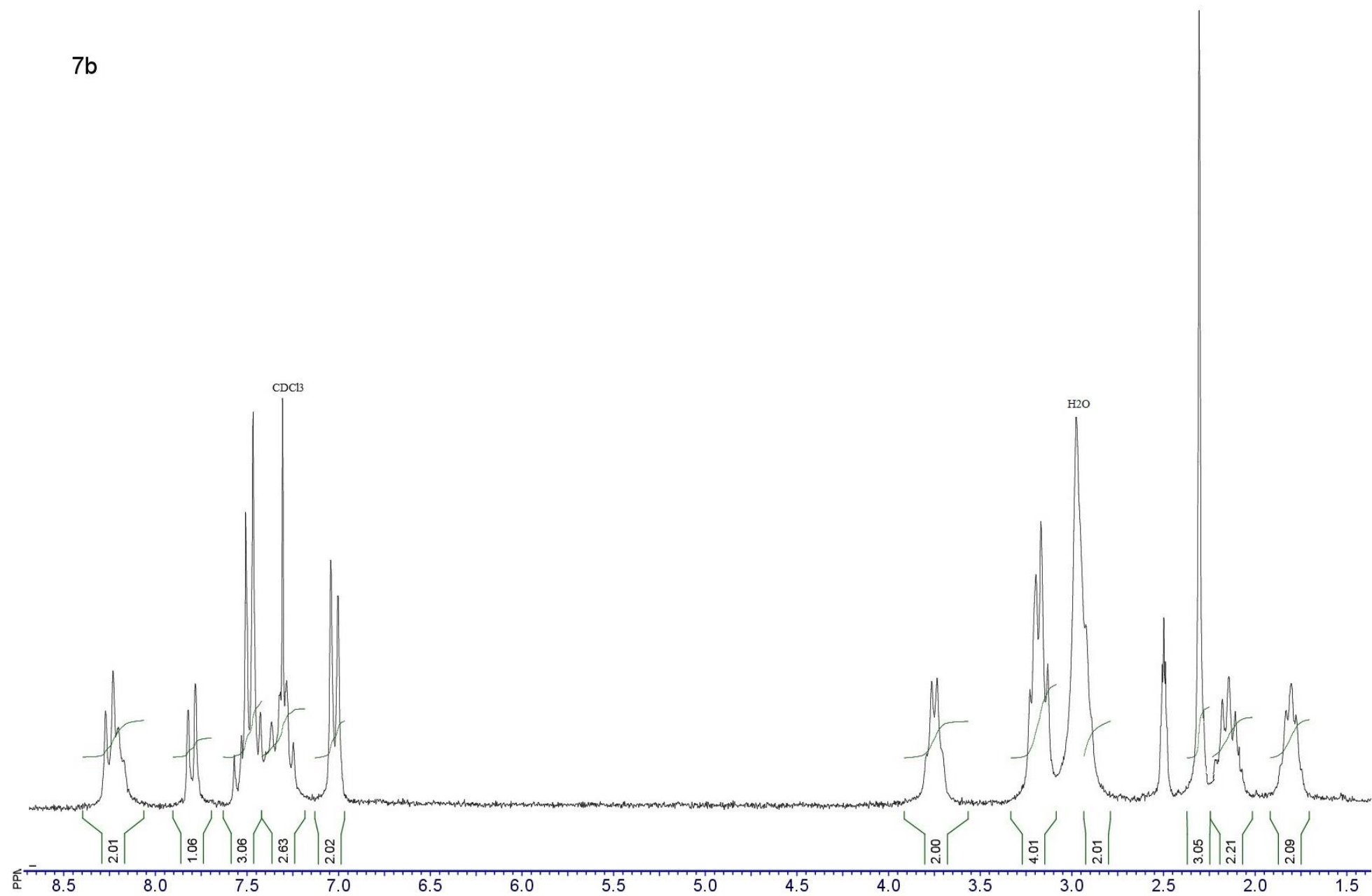

7c

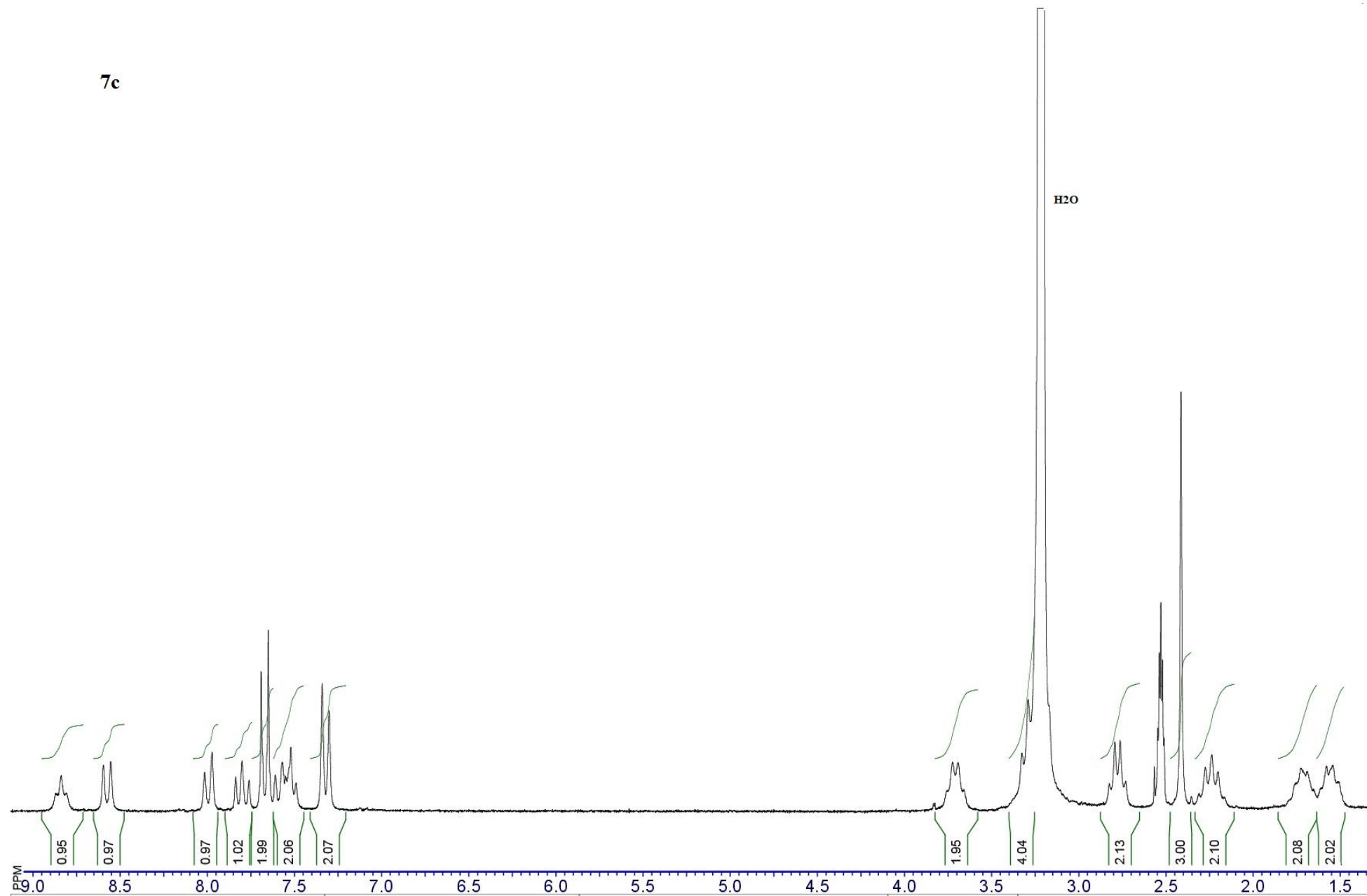

7d

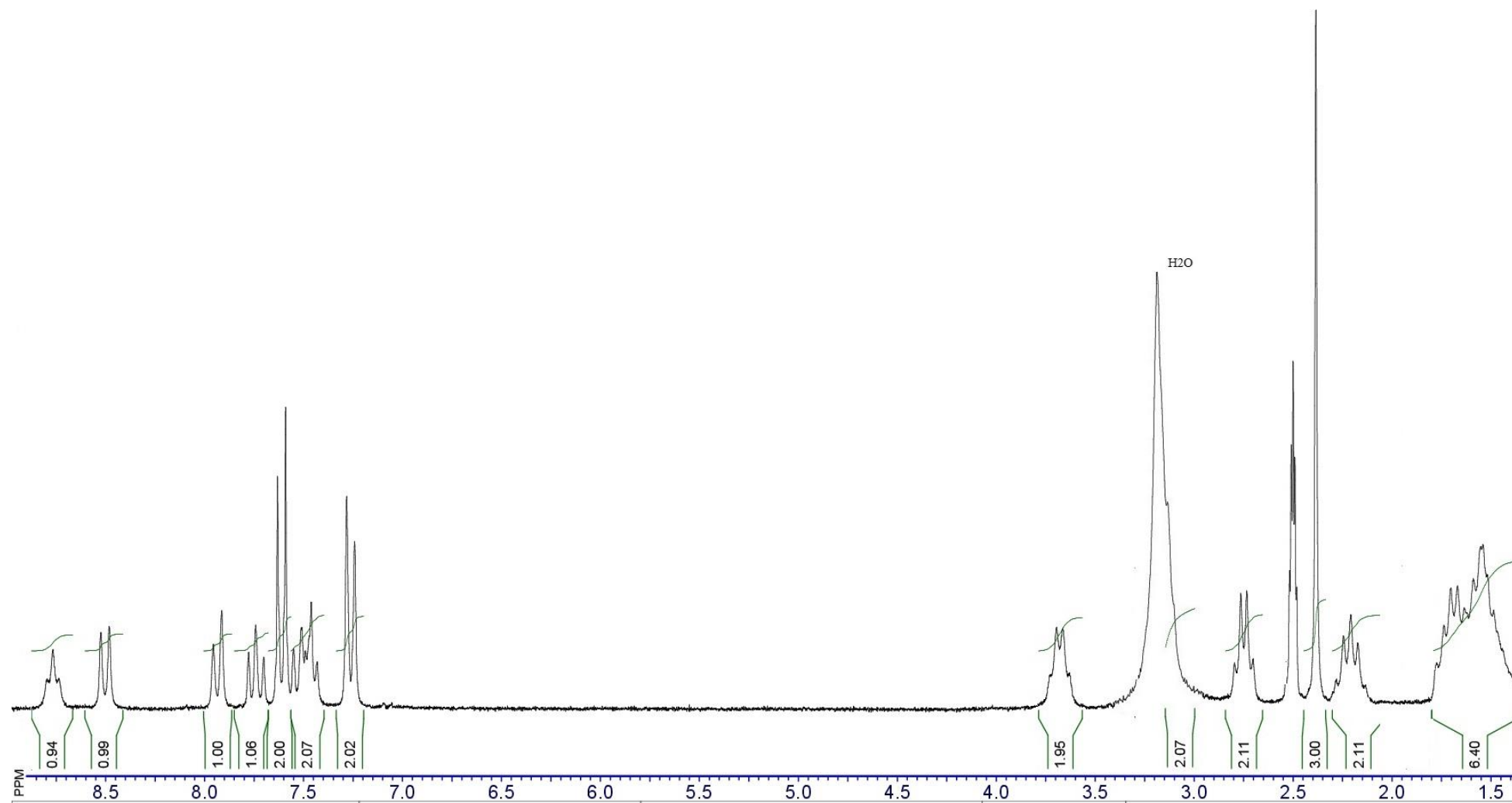

7e

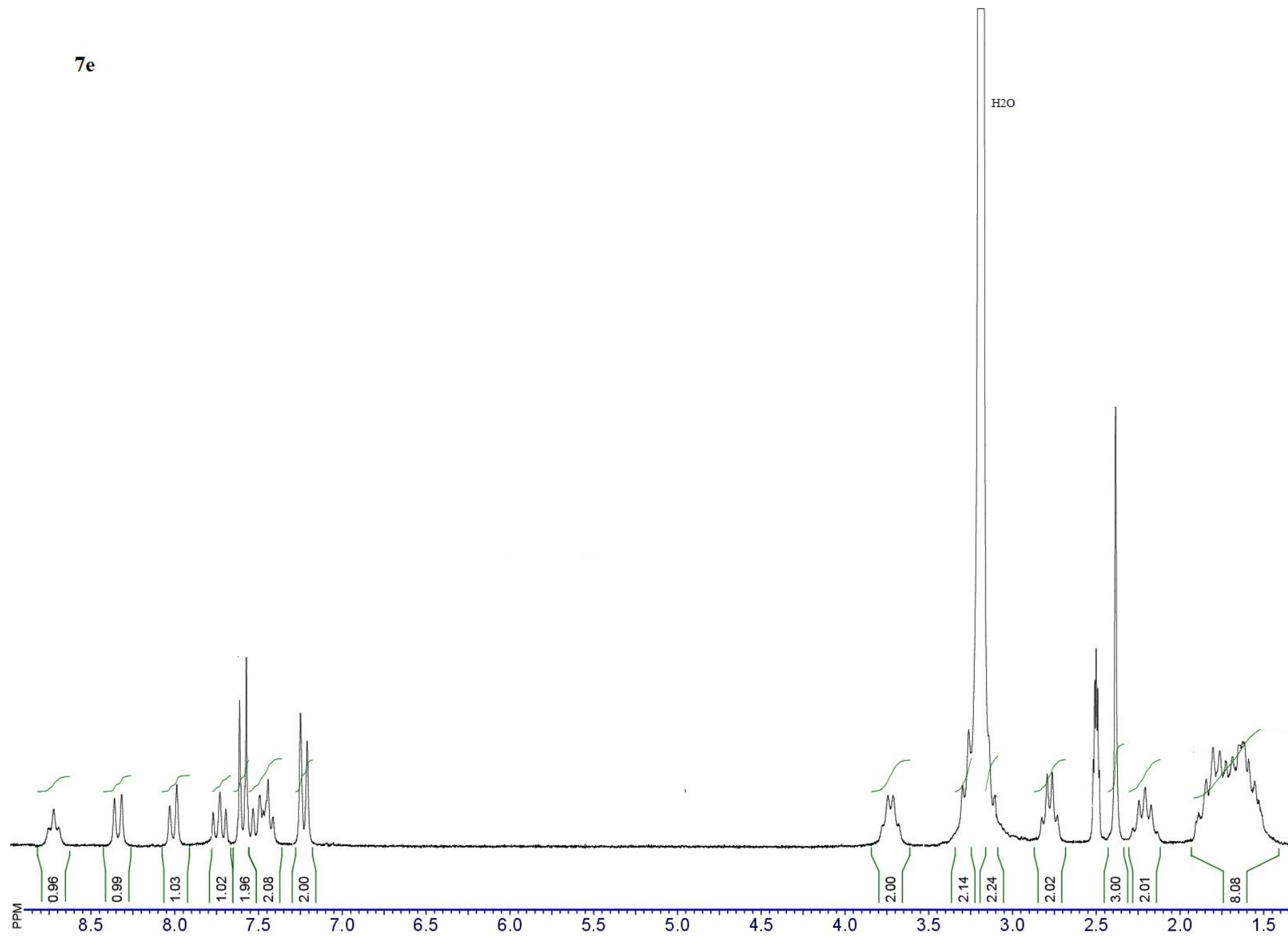

7f

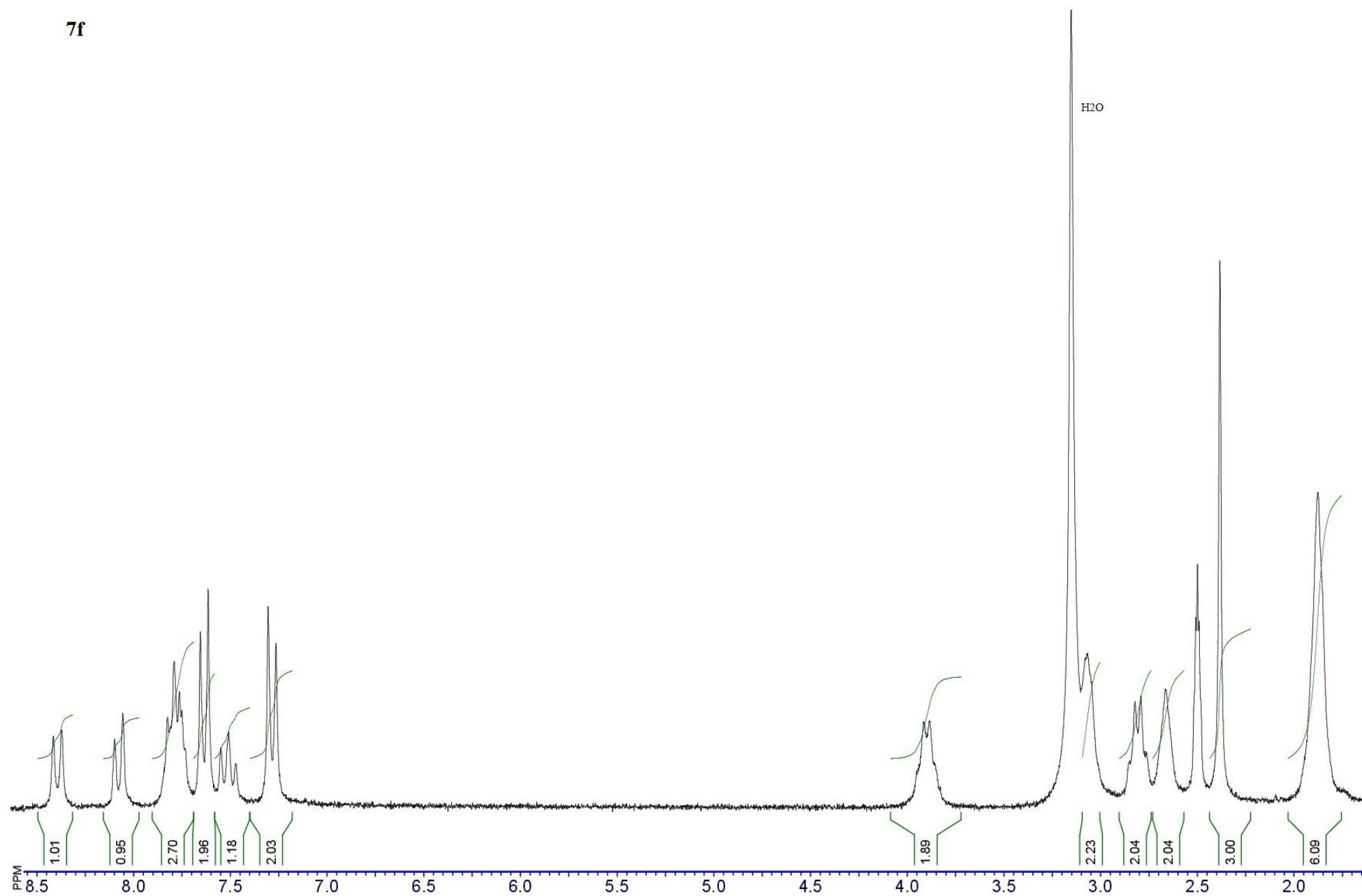

7g

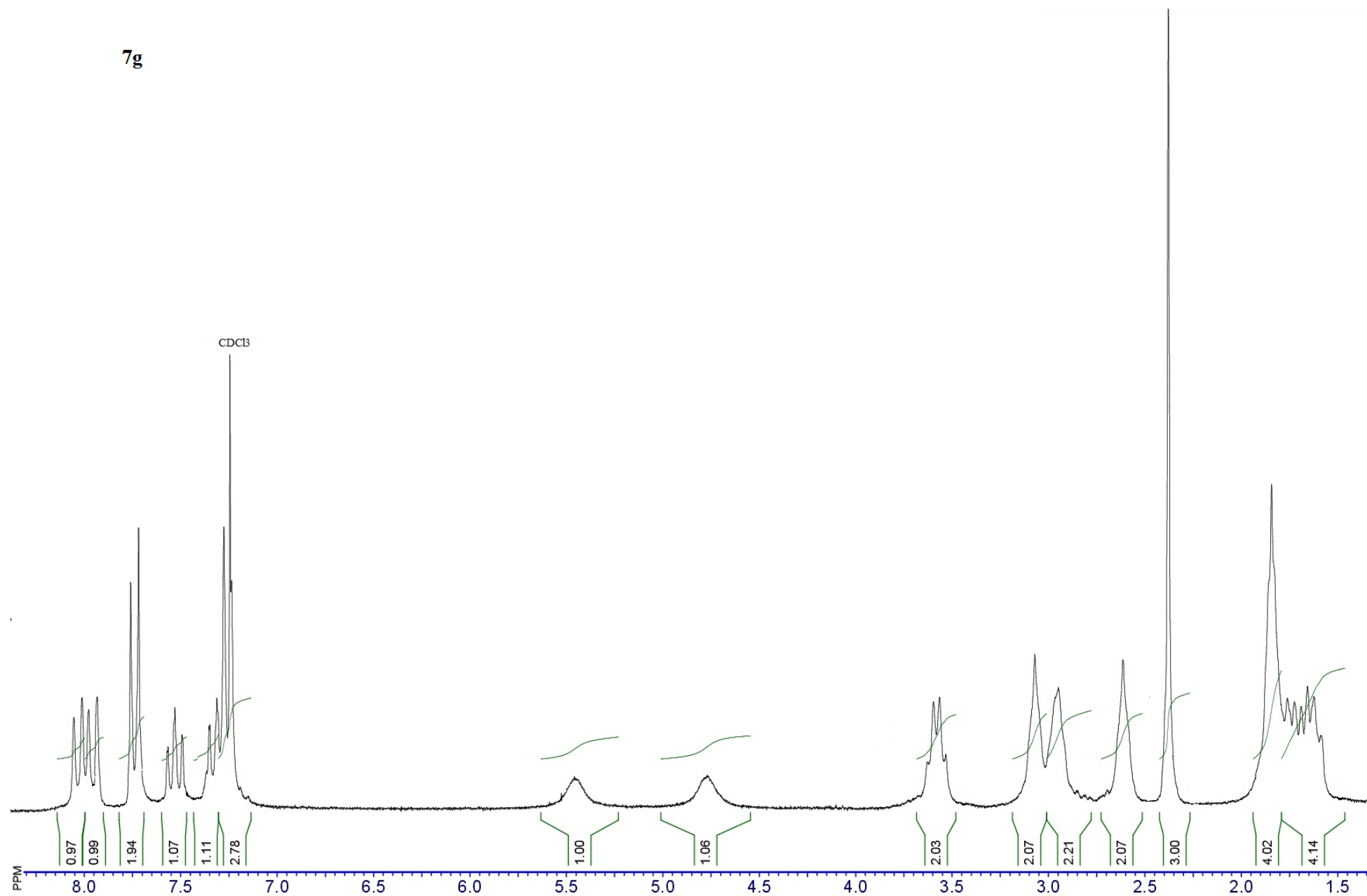

7h

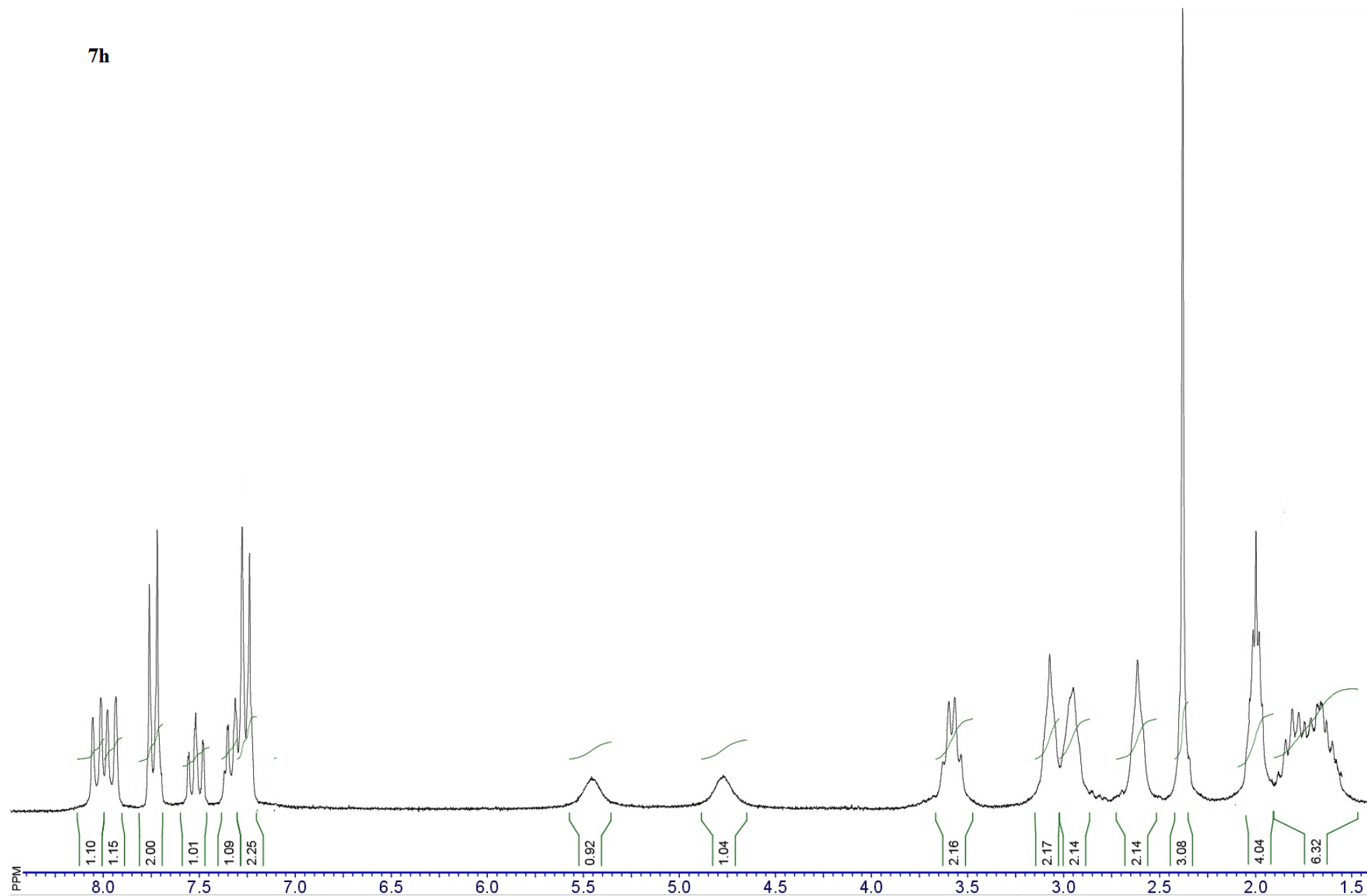

7i

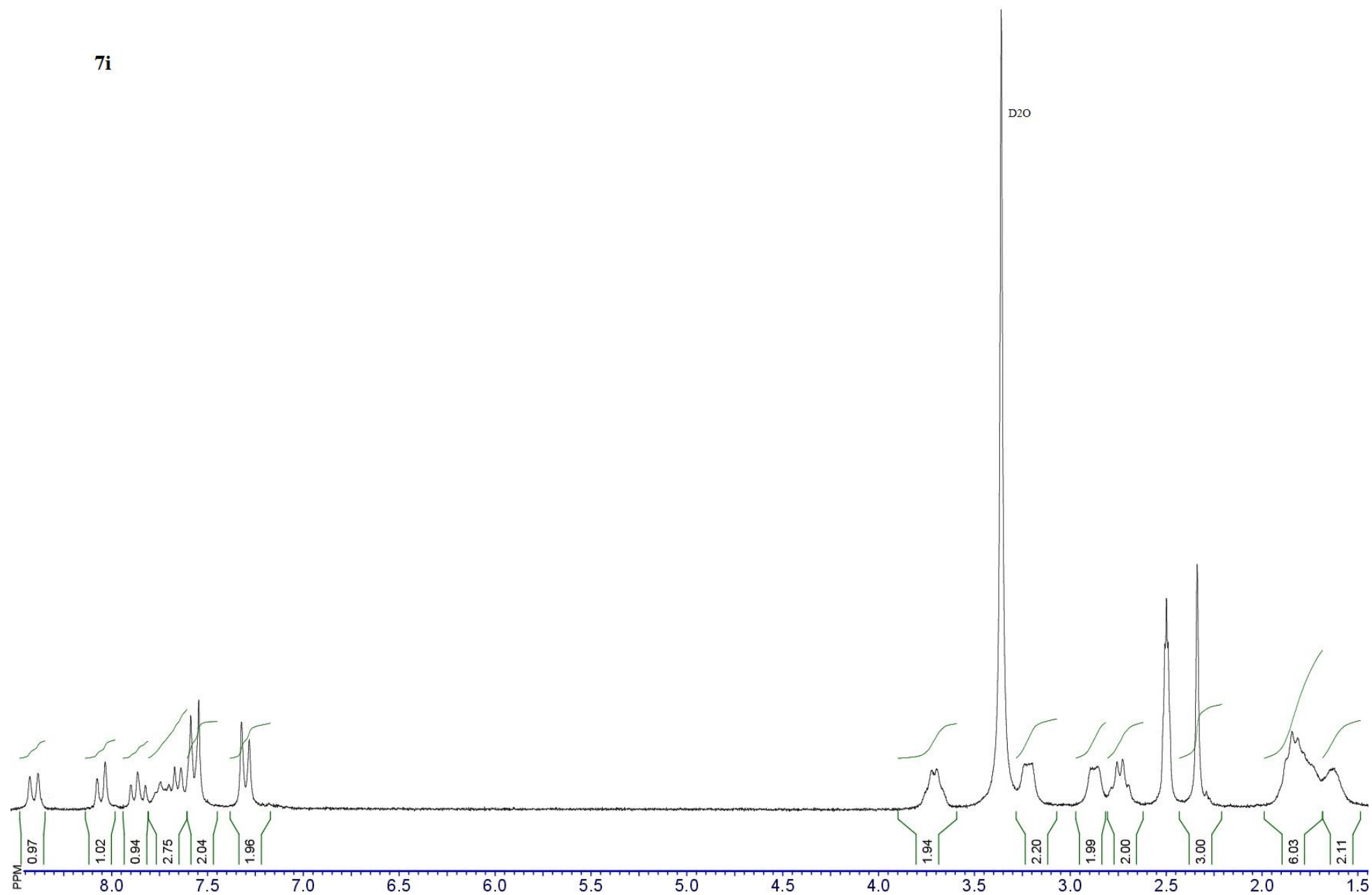

7j

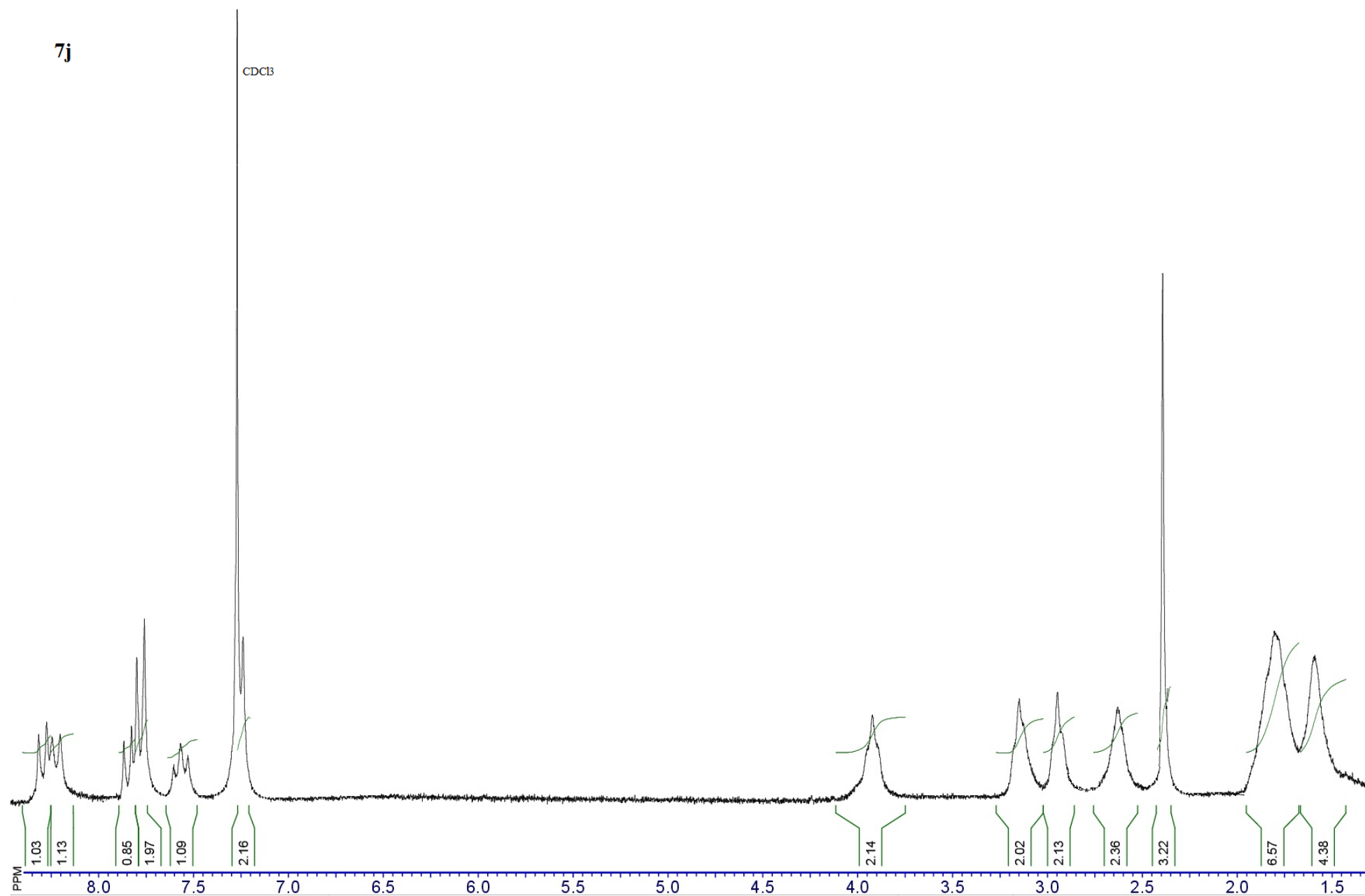

7k

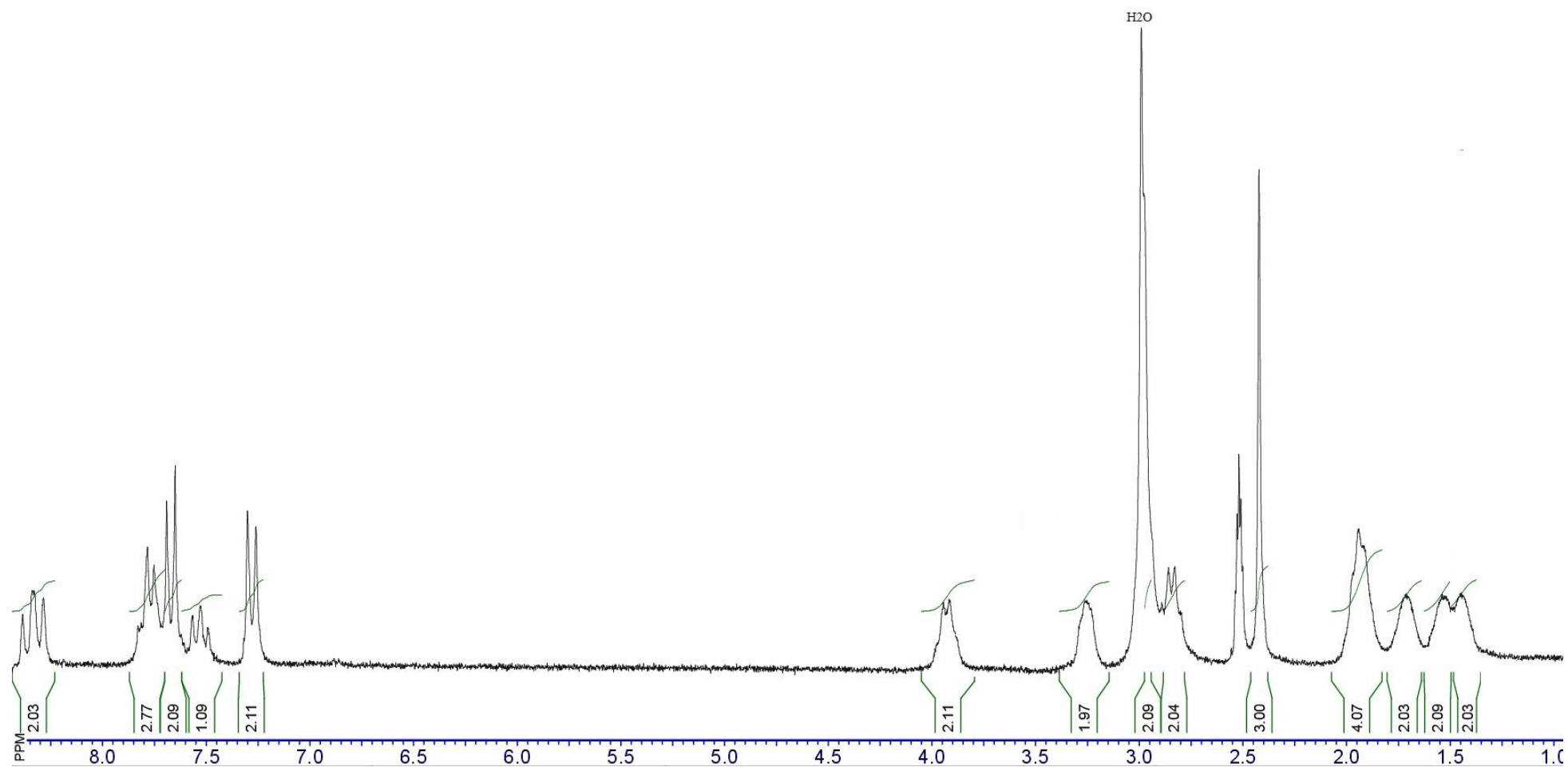

Supplement: Supplementary file 1 [file molecules-25-03915-s001.zip › Supplementary_NMR_spectra.pdf]
